# A novel water‐soluble near‐infrared fluorescent probe for monitoring viscosity fluctuations in plants and zebrafish under abiotic stresses

**DOI:** 10.1002/smo2.70043

**Published:** 2026-03-19

**Authors:** Yixue Feng, Keke Ding, Yongxu Yang, Quanliang Liu, Silin Xu, Chunhui Liu, Zhihong Xu, Xufeng Hou, Jihong Huang, Tony D. James

**Affiliations:** ^1^ Key Laboratory of Chemo/Biosensing and Detection of Xuchang Key Laboratory of Micro‐Nano Materials for Energy Storage and Conversion of Henan Province Henan Joint International Research Laboratory of Nanomaterials for Energy and Catalysis College of Chemical and Materials Engineering Xuchang University Xuchang China; ^2^ College of Chemistry Zhengzhou University Zhengzhou China; ^3^ School of Science Xuchang University Xuchang China; ^4^ Collaborative Innovation Center of Functional Food by Green Manufacturing Food and Pharmacy College Xuchang University Xuchang China; ^5^ Department of Chemistry University of Bath Bath UK; ^6^ School of Chemistry and Chemical Engineering Henan Normal University Xinxiang China

**Keywords:** biocompatibility, large Stokes shift, near infrared probe, plant imaging, viscosity sensing

## Abstract

Viscosity is one of the important microenvironmental parameters of abiotic stress. Abnormal changes in viscosity have been shown to be correlated with pathological activities and diseases of plants. However, current probes may not be suitable for sensing in plants due to their hydrophobicity and the phytochromes found in plants. With this research, we successfully constructed a structurally simple, water‐soluble near‐infrared fluorescent probe HJA‐MQ‐D, which can simultaneously monitor dynamic viscosity changes in plants and animal systems. The probe exhibits a relatively long emission wavelength (nearly 700 nm) and large Stokes shift (88 nm), and exceptional viscosity‐responsive performances with 140‐fold fluorescence intensity enhancement. More importantly, the HJA‐MQ‐D can image the viscosity fluctuation at the cellular level in plants (onion inner epidermis and mung bean sections) as well as at the whole‐plant level (mung bean and peanut seedling). In addition, HJA‐MQ‐D was used to monitor the viscosity changes in a nystatin‐induced zebrafish model. Based on these advanced characteristics, we anticipate that this probe will provide a powerful new tool for cross‐species viscosity monitoring.

## Graphical abstract

HJA‐MQ‐D exhibits a rapid and sensitive response to viscosity changes based on the twisted intramolecular charge transfer mechanism. Displaying near‐infrared emission and excellent water‐solubility, enabling imaging at the cellular and whole plant level.

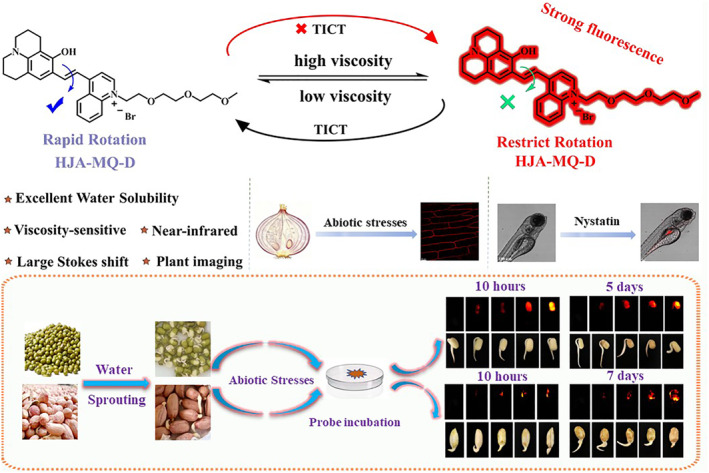

## 1 INTRODUCTION

Plants are an indispensable resource found on the earth, and play a crucial role in maintaining human survival, facilitating the carbon cycle, promoting biodiversity, and enhancing the quality of the ecosystem.[^1^, ^2^] However, due to the enhanced industrial development of humans resulting in extreme meteorological conditions, plants, especially crops, are more regularly subjected to various adverse abiotic stresses, such as drought, salinity, temperature fluctuations, waterlogging and heavy metal contamination, which hamper plant growth, resulting in plant species becoming endangered, and inducing a decline in agricultural productivity.[^3^, ^4^, ^5^, ^6^, ^7^, ^8^] The abiotic stress response of plants represents a highly complicated biological process, and the underlying molecular mechanisms remain unknown.^[9]^ Therefore, the early and precise monitoring of abiotic stress in plants is an effective method to ensure efficient plant growth, maintain plant health, and enhance crop yields. Research has shown that some reactive species such as reactive oxygen species (ROS), reactive nitrogen species, reactive sulfur species, enzymes and microenvironmental parameters (viscosity, polarity, pH) etc., are dynamically balanced in plants.[^10^, ^11^] However, this balance can be disrupted when plants undergo abiotic stresses, resulting in fluctuations of these reactive species in plants.[^12^, ^13^, ^14^, ^15^, ^16^, ^17^, ^18^, ^19^, ^20^, ^21^, ^22^, ^23^, ^24^, ^25^] As such, these species can be used as biomarkers for the degree of abiotic stresses in plants. Due to these aspects, it is imperative to devise a platform for the real‐time, rapid and in situ monitoring of fluctuations in reactive species within plants in order to understanding more deeply plant physiological processes and mechanisms.[^20^, ^26^, ^27^, ^28^, ^29^]

Viscosity, as an extremely important microenvironmental parameter, plays a vital role in maintaining normal physiological functions in living organisms, where variations are closely associated with abiotic stresses in plants, affecting growth, development and physiological functions, such as pathogenic infections that result in sticky sap hindering transportation and drought stress that increases cytoplasmic viscosity interfering with the metabolism.[^30^, ^31^, ^32^] Although numerous viscosity detection methods have been developed, such as capillary viscometers, rotational viscometers, ultrasonic viscometers, falling‐ball viscometers, and vibrational viscometers,[^33^, ^34^, ^35^, ^36^] these analytical techniques cannot be applied for monitoring viscosity changes in living organisms. As such, fluorescence‐based imaging has emerged as an invaluable tool for biomedical research in living organisms,[^37^, ^38^, ^39^, ^40^, ^41^, ^42^, ^43^, ^44^, ^45^, ^46^] due to numerous advantages, such as easy operation, high sensitivity, selectivity, non‐invasive monitoring, and real‐time imaging. Among various fluorescent probes, near‐infrared (NIR) fluorescent probes exhibit superior imaging capabilities due to their long‐wavelength emission (650–900 nm) (Table S1). This window endows the fluorescent probes with outstanding advantages for imaging reactive species in plants, such as enhanced tissue transparency, deeper tissue penetration, weaker tissue absorption and scattering, reduced phototoxicity and chlorophyll autofluorescence interference.[^47^, ^48^, ^49^, ^50^, ^51^, ^52^, ^53^, ^54^, ^55^, ^56^, ^57^] In recent years, several methods have been used to develop novel NIR fluorophores: increasing the electron‐donating/electron‐withdrawing ability of the electron‐donating/electron‐withdrawing groups and extending π‐conjugation. These strategies have significantly reduced the water solubility of the fluorophore. Meaning that these probes work better in animal systems, and can not be used in plant systems due to the high hydrophobicity. As such, the design of a water‐soluble NIR probe for real‐time, rapid and in situ monitoring of the change in viscosity in plants is essential.

The construction of NIR fluorescent probes for viscosity monitoring is primarily based on twisted intramolecular charge transfer (TICT). Such probes typically consist of a fluorophore moiety and an acceptor group connected through a double bond. In different viscosity environments, the fluorescence intensity depends on the rotational freedom of the entire fluorophore‐acceptor moiety. In low‐viscosity systems, the rapid rotation results in weak fluorescence intensity, whereas in high‐viscosity systems, the restricted rotation leads to strong fluorescence emission. Based on this principle, we chose 8‐hydroxyjulolidine as the fluorescent group to make the emission wavelength as close as possible to the NIR wavelength (Table S2). By combining it with the quinoline triethylene glycol monomethyl ether cation through a double bond, a novel NIR viscosity‐sensitive fluorescent probe HJA‐MQ‐D was synthesized. The introduction of the quinoline group enhances the conjugated structure of HJA‐MQ‐D. Meanwhile, the quinoline cation and triglycerol monomethyl ether significantly improve its water solubility and cell membrane permeability. Just as we expected, compared with previously reported viscosity probes, HJA‐MQ‐D exhibits superior water solubility, enhanced photostability and wider applicability, and can dynamically monitor the viscosity changes of various model organisms (including plant and animal cells) in real time, and as such is expected to be a powerful tool for cross‐species viscosity monitoring research.

## 2 EXPERIMENTAL SECTION

### 2.1 Synthesis of HJA‐MQ‐D

**SCHEME 1:**
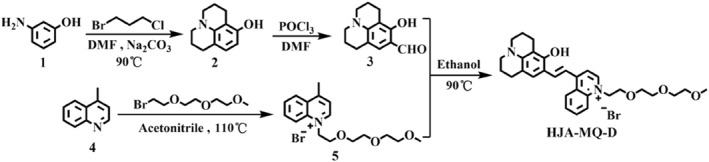
Synthetic route for HJA‐MQ‐D.

The synthetic route for the probe HJA‐MQ‐D is shown in Scheme 1.

*Compound 2, compound 3, and compound 5*: The raw materials for synthesizing compound 2 are *m*‐aminophenol and 1‐Bromo‐3‐chloropropane. Compound 3 was synthesized by reacting compound 2 with phosphorous oxybromide in dimethylformamide (DMF). The raw materials for synthesizing compound 5 are 4‐methyl quinoline and diethylene glycol 2‐bromoethyl methyl ether. Compound 2, 3, and 5 were synthesized based on the previously reported literature.[^58^, ^59^] The detailed synthetic procedures and corresponding structural characterization are depicted in the Supporting Information (Scheme S1 and Figures S1–S6).

*HJA‐MQ‐D*: Compound 3 (123.13 mg, 0.567 mmol) and compound 5 (209.95 mg, 0.567 mmol) were dissolved in anhydrous ethanol (10 mL). The mixture was the refluxed at 90°C for 14 h. Upon cooling to room temperature, the crude product was concentrated under reduced pressure and subsequently purified by preparative thin‐layer chromatography with a mixed solvent system of dichloromethane/methanol (50:1 v/v) as eluent. The final product HJA‐MQ‐D was obtained and collected as a deep blue solid (102 mg, 32% yield). ^1^H NMR (400 MHz, Methanol‐d4, Figure S7) *δ* 8.45 (d, *J* = 8.6 Hz, 1H), 8.40 (d, *J* = 6.9 Hz, 1H), 8.08 (d, *J* = 15.2 Hz, 1H), 8.00 (d, *J* = 8.8 Hz, 1H), 7.88–7.82 (m, 1H), 7.68–7.61 (m, 2H), 7.44 (d, *J* = 15.1 Hz, 1H), 7.16 (s, 1H), 4.70 (t, *J* = 4.8 Hz, 2H), 3.85 (t, *J* = 4.7 Hz, 2H), 3.49 (dd, *J* = 5.6, 2.9 Hz, 2H), 3.43–3.40 (m, 2H), 3.35 (dd, *J* = 6.0, 3.3 Hz, 2H), 3.31–3.28 (m, 2H), 3.21 (dt, *J* = 4.0, 2.0 Hz, 4H), 3.17 (s, 3H), 2.61 (t, *J* = 6.2 Hz, 2H), 2.51 (t, *J* = 6.4 Hz, 2H), 1.84 (t, *J* = 6.1 Hz, 4H). ^13^C NMR (100 MHz, Methanol‐d4, Figure S8) *δ* 155.26, 154.41, 148.35, 145.08, 141.30, 138.46, 133.92, 127.49, 126.62, 126.06, 125.82, 117.85, 116.00, 112.75, 111.10, 109.75, 106.65, 71.51, 70.32, 70.08, 69.91, 67.94, 57.71, 55.05, 50.04, 49.25, 27.05, 21.58, 20.77, 20.67. ESI‐MS (m/z) for C_30_H_37_N_2_O_4_^+^ [M]^+^: Calcd 489.2748, found 489.2740 (Figure S9).

### 2.2 Imaging of plant

*Imaging of onion epidermal cells*: Using onion inner epidermal cells as an experimental model, firstly, onion inner epidermal tissues were peeled off and stained using probe HJA‐MQ‐D, then different models were constructed to change the environmental viscosity,^[25]^ and finally confocal fluorescence imaging was performed.

*Imaging of mung bean seedling slices*: Sliced mung bean seedling cells were used as the experimental model; firstly, mung bean seeds were cultured in water until germination, and then mung bean seedling stem tissue sections were prepared.^[60]^ The slices were labeled with probe HJA‐MQ‐D, and then the viscosity of the cellular microenvironment was changed using a sucrose solution, and finally confocal fluorescence microimaging was performed.

*Imaging of whole plants*: In order to systematically evaluate the tracking performance of probe HJA‐MQ‐D in plant rhizome systems, a comparative model based on mung bean and peanut was established.[^47^, ^61^, ^62^, ^63^] By setting two treatment conditions, heavy metal and salt stress, an in vivo imaging system was used for dynamic monitoring.

### 2.3 Imaging of zebrafish

All animal protocols adhered to the care and usage guidelines established by Xuchang University for laboratory animals. The experiments were sanctioned by the Xuchang University Medical Ethics Committee (Henan, China, ethics statement Reference No. 2025014). Zebrafish were purchased from Nanjing EzeRinka Biotechnology Co., Ltd., Zebrafish (5 days old) larvae were first pre‐incubated with 10 μM Nystatin (Nys) for 30 min in a culture medium at 28°C. Subsequently, probe HJA‐MQ‐D 10 μM was added and incubated for a total of 30 min. After incubation, wash three times (1 min/time) with fresh culture media (pre equilibrated at 28°C) to remove unbound probes and metabolites, and finally perform in vivo imaging using confocal microscopy.^[19]^

## 3 RESULTS AND DISCUSSION

### 3.1 Comparison of water solubility of probes

**FIGURE 1:**
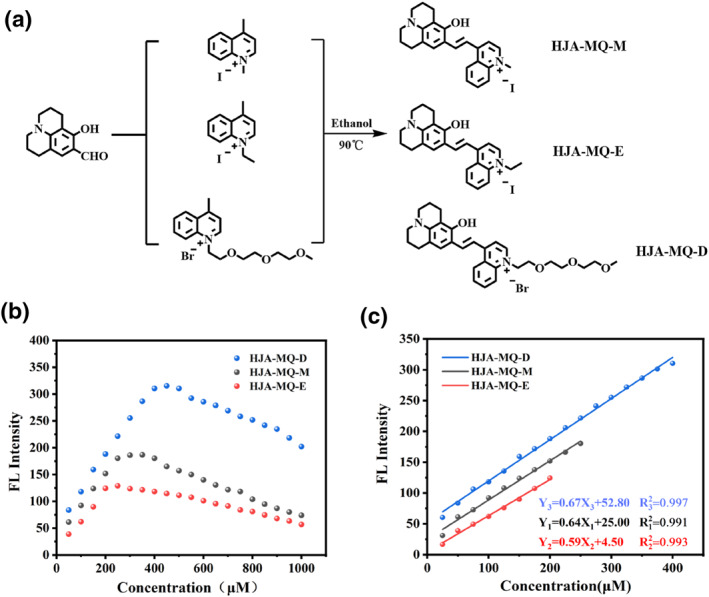
(a) Chemical structures of HJA‐MQ‐M, HJA‐MQ‐E and HJA‐MQ‐D. (b) Fluorescence intensity versus concentration curves of the three probes (solvent: H_2_O). (c) Linear fitting results of fluorescence intensity versus concentration in the low concentration region (0–400 μM) for the three probes. The excitation wavelength was 610 nm. Slits = 10/10 nm.

In order to design fluorescent probes with better water solubility, we introduced methyl, ethyl and triethylene glycol monomethyl ether groups onto quinoline to synthesize three different structures of probes, namely HJA‐MQ‐M, HJA‐MQ‐E, and HJA‐MQ‐D (Figure 1a). The corresponding synthetic methods and structural characterization data can be found in the Supporting Information, as shown in Figures S10–S13 and Scheme S2. These probes were prepared as aqueous solutions of different concentrations and subjected to fluorescence intensity experiments. The results indicated that HJA‐MQ‐M, HJA‐MQ‐E, and HJA‐MQ‐D reached their maximum fluorescence intensity at concentrations of 250, 200, and 400 μM, respectively (Figure 1b), indicating that HJA‐MQ‐D had the best water solubility (Table S3). At the same time, we also analyzed the linear relationship between fluorescence intensity and concentration of each probe from low concentration to the fluorescence peak concentration. All probes exhibited good linear responses (*R* ^2^ > 0.99) (Figure 1c), confirming that the fluorescence intensity is proportional to the concentration in the monomer state. The wide linear range of HJA‐MQ‐D further supported its excellent water solubility and practicality.

### 3.2 Spectral response of HJA‐MQ‐D

*Sample treatment*: HJA‐MQ‐D was dissolved in DMSO to prepare a 1 mM stock solution, which was then dispensed into light‐proof containers and stored at −30°C in the dark to ensure long‐term stability.

**FIGURE 2:**
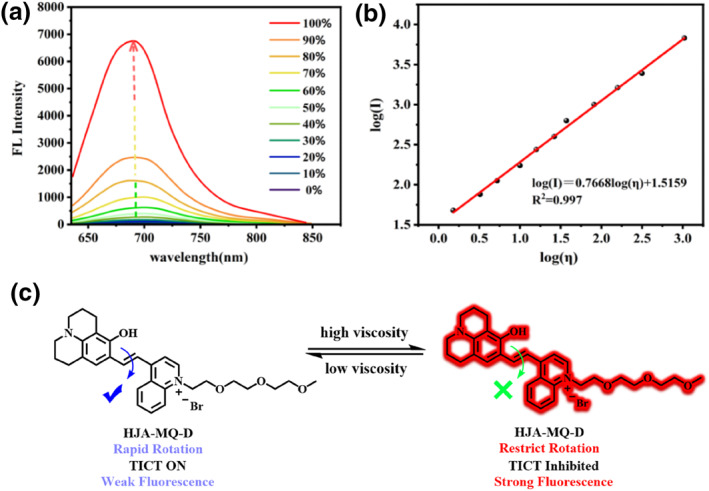
(a) Fluorescence spectra of probe HJA‐MQ‐D (10 μM) in glycerol‐PBS mixed solutions with different viscosities (*λ*_ex_ = 610 nm, slits = 10/10 nm). (b) Linear relationship between probes log *I*_697.6_ and log *η*. (c) Structure and response mechanism toward viscosity for HJA‐MQ‐D. The excitation wavelength was 610 nm. Slits = 10/10 nm.

*Response of HJA‐MQ‐D toward viscosity*: Based on the characteristics of HJA‐MQ‐D such as simplicity of synthesis, good water solubility, and excellent biocompatibility, as well as the TICT properties due to the presence of double bonds between 8‐hydroxyjulolidine and quinoline cation derivatives, we systematically investigated the spectral response behavior of the probe HJA‐MQ‐D in different viscosity solutions (0%–100% glycerol‐phosphate buffered saline (PBS)). The experimental results indicated that the UV absorption spectrum of the probe HJA‐MQ‐D exhibited a significant redshift with increasing glycerol percentage, with the maximum absorption peak shifting from 606 to 638 nm (Figure S14). A sharp enhancement of fluorescence intensity at 697.6 nm with increasing viscosity was observed in the fluorescence spectra, and the fluorescence intensity at the highest viscosity condition reached 140‐fold that at the lowest viscosity (Figure 2a). This is because in the low viscosity solution, the rotor can rotate freely, which inhibits the fluorescence emission to a certain extent. However, in the high viscosity solution, the rotation of the rotor is restricted, and the energy could only be released in the form of fluorescence (Figure 2c). Further quantitative analysis indicated that there was a good linear correlation between log *I*_697.6_ and log *η* (Figure 2b), and these results fully confirmed the highly sensitive response characteristics of the HJA‐MQ‐D probe to changes in media viscosity.

**FIGURE 3:**
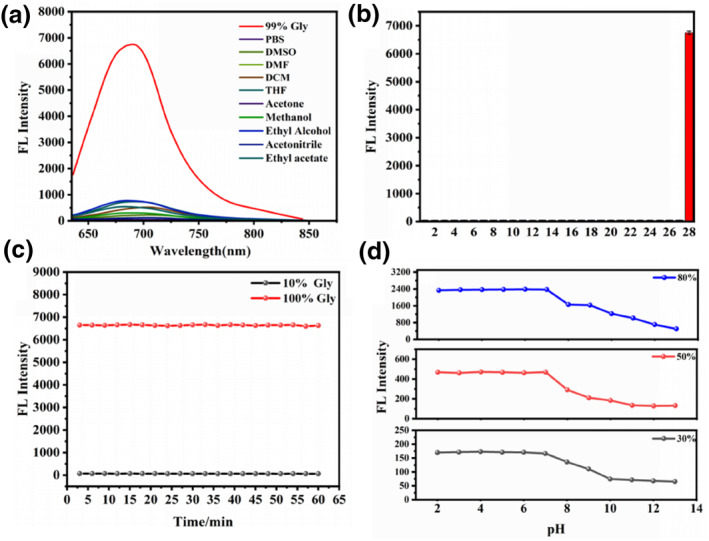
(a) Fluorescence spectra of probe HJA‐MQ‐D (10 μM) in different solvents. (b) Fluorescence spectra of probe HJA‐MQ‐D in response to glycerol and 20 μM other analytes (1. Zn^2+^, 2. Ni^2+^, 3. K^+^, 4. Co^2+^, 5. Ba^2+^, 6. Mn^2+^, 7. Fe^3+^, 8. Cu^2+^, 9. Cd^2+^, 10. Na^+^, 11. Ca^2+^, 12. N_2_H_4_, 13. S^2−^, 14. F^−^, 15. Cl^−^, 16. Br^−^, 17. ClO^−^, 18. HSO_3_^−^, 19. Proline, 20. Glutamic acid, 21. Lysine, 22. Arginine, 23. Tryptophan, 24. Phenylalanine, 25. Cysteine, 26. Glutathione, 27. Homocysteine, 28. Glycerol (99%)). (c) Stability of probe HJA‐MQ‐D in high viscosity and low viscosity environments. (d) Fluorescence spectra of probe HJA‐MQ‐D versus pH at different viscosities (glycerol‐PBS mixture with different proportions). The excitation wavelength was 610 nm. Slits = 10/10 nm.

To systematically evaluate the specific response of the probe HJA‐MQ‐D to viscosity, we carried out a series of control experiments. First, by measuring the fluorescence spectra of the probe in different polar solvents (including dimethyl sulfoxide (DMSO), DMF, dichloromethane, tetrahydrofuran, acetone, methanol, ethyl alcohol, acetonitrile and ethyl acetate) (Figure 3a), it was found that the solvent polarity had a minimal effect on the fluorescence response of the probe HJA‐MQ‐D. In order to investigate the response of the probe HJA‐MQ‐D to common interfering substances in the cell, we evaluated the fluorescence spectra of the probe HJA‐MQ‐D in a PBS system with different cations, anions and amino acids (1. Zn^2+^, 2. Ni^2+^, 3. K^+^, 4. Co^2+^, 5. Ba^2+^, 6. Mn^2+^, 7. Fe^3+^, 8. Cu^2+^, 9. Cd^2+^, 10. Na^+^, 11. Ca^2+^, 12. N_2_H_4_, 13. S^2−^, 14. F^−^, 15. Cl^−^, 16. Br^−^, 17. ClO^−^, 18. HSO_3_^−^, 19. Proline, 20. Glutamic acid, 21. Lysine, 22. Arginine, 23. Tryptophan, 24. Phenylalanine, 25. Cysteine, 26. Glutathione, 27. Homocysteine, 28. Glycerol (99%)), the results indicated that the probe exhibited good selectivity for glycerol (Figure 3b) and negligible interference from the above substances (Figure S15). The probe HJA‐MQ‐D maintained stable fluorescence performance under both low and high viscosity conditions (Figure 3c). In addition, the effect of pH on the performance of probe HJA‐MQ‐D was systematically investigated by constructing a glycerol‐PBS (30%, 50%, 80%) mixed solvent system over a pH range 2 to 13. The experimental results indicated that the fluorescence intensity of the probe HJA‐MQ‐D maintains stability over physiologically relevant pH range (2–7) (Figure 3d), confirming excellent pH tolerance.

### 3.3 Theoretical calculations

**FIGURE 4:**
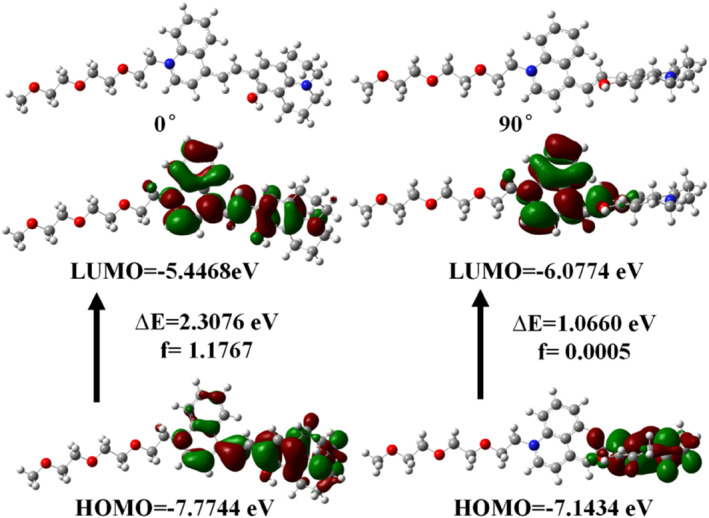
Computational analysis of HJA‐MQ‐D: geometrical structures optimized for both ground and excited states; Frontier molecular orbitals corresponding to a 0° dihedral angle in the excited state; Frontier molecular orbitals at a 90° dihedral angle in the excited state. (All calculations conducted with Gaussian 16 at the PBE0/6‐311G (d, p) theoretical level.)

This study employed systematic theoretical simulations of the probe molecule HJA‐MQ‐D using Gaussian 16 software to investigate its configuration‐dependent electronic structure characteristics and optical behavior. Geometric optimization of the molecule was performed based on density functional theory (DFT) and time‐dependent (TD)‐DFT, and the energy level distribution of the frontier molecular orbitals was calculated. The results indicate that the molecule exhibits an optimal structure in the excited state when the 8‐hydroxyjulolidine and the quinoline part adopt a nearly coplanar conformation. In this configuration, the dihedral angle between the two segments is 0°, the charge distribution becomes more uniform, and the spatial overlap between the highest occupied molecular orbital (HOMO) and lowest unoccupied molecular orbital (LUMO) electron clouds is significantly enhanced. This substantially increases the fluorescence oscillator strength (*f* = 1.1767). This indicates that the molecule exhibits excellent fluorescence emission capability in this planar configuration, with a HOMO‐LUMO energy gap of 2.3076 eV. However, when the dihedral angle increases to 90°, the molecule transitions to a twisted charge‐transfer state (TICT state). In this state, the LUMO electron density is primarily concentrated in the quinoline part, while the HOMO is localized in the julolidine unit, reducing the orbital energy gap to 1.0660 eV. Due to the significant spatial separation between HOMO and LUMO, the orbital overlap is substantially reduced, leading to a sharp decrease in the fluorescence oscillator strength (*f* = 0.0005). Fluorescence emission is strongly suppressed, and molecular energy is primarily dissipated through nonradiative transition pathways. These results demonstrate that the luminescence behavior of HJA‐MQ‐D strongly depends on the molecular conformation: the planar conformation favors radiative transitions, while the TICT state promotes nonradiative decay. This conformation‐sensitive fluorescence characteristic enables it to efficiently respond to changes in microenvironmental viscosity, making it a promising viscosity‐sensitive fluorescent probe (Figure 4).

### 3.4 Viscosity detection in plants

*Plant cultivation protocols and evaluation conditions*: Plant cell viscosity is modulated by multiple factors, including environmental stresses (drought, high salinity, heavy metals, and oxidative stress), biotic stresses (pathogen infection), developmental stages (cell division and differentiation), and metabolic activities (ROS bursts and cytoskeletal reorganization). Among environmental stresses, cytoplasmic gelation significantly increases viscosity, with specific manifestations including protein aggregation caused by high salt concentrations, disulfide bond crosslinking promoted by oxidative stress (H_2_O_2_), and redox homeostasis disruption by heavy metals (Cd^2+^). In order to systematically assess this effect, we constructed different models for evaluating and quantifying the relative fluorescence intensity of the red channel with all experiments performed in triplicate to ensure data reliability. To validate the probe's response specificity in complex biological environments, orthogonal control experiments simulating macromolecular crowding and protein enrichment systems were conducted. Through systematic evaluation and exclusion of non‐specific protein binding interference, the probe's fluorescence enhancement signal was confirmed to exhibit high selectivity toward viscosity changes (Table S4, Figures S16–S18). Cellular imaging was conducted using a Leica DMI 8 laser scanning confocal microscope with an excitation wavelength of 638 nm and a detection range of 680–730 nm.

**FIGURE 5:**
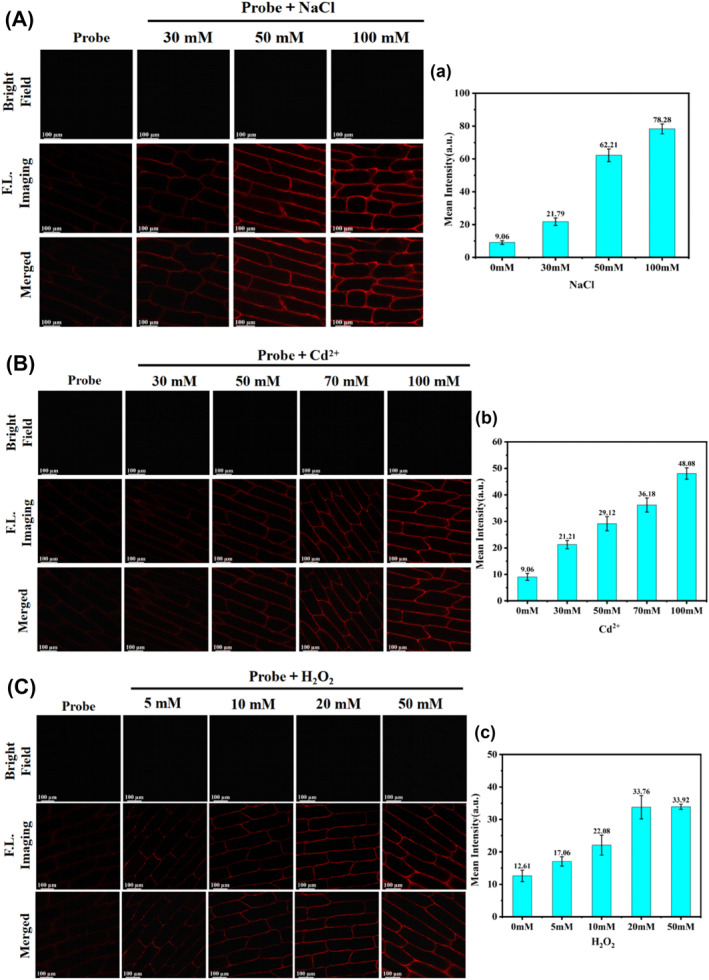
(A) Onion inner epidermis stained with 5 μM of fluorescent probe HJA‐MQ‐D for 5 min, then treated with different NaCl concentrations (0, 30, 50, 100 mM) for 5 min. (a) Quantitative analysis of relative fluorescence intensity in the red channel for panel (A). (B) Onion inner epidermis stained with 5 μM of fluorescent probe HJA‐MQ‐D for 5 min, then treated with different Cd^2+^ concentrations (0, 30, 50, 70, 100 μM) for 5 min. (b) Quantitative analysis of relative fluorescence intensity in the red channel for panel (B). (C) Onion inner epidermis stained with 5 μM of fluorescent probe HJA‐MQ‐D for 5 min, then treated with different H_2_O_2_ concentrations (0, 5, 10, 20, 50 mM). (c) Quantitative analysis of relative fluorescence intensity in the red channel for panel (C). Red channel: *λ*_ex_ = 638 nm, *λ*_em_ = 680–730 nm, scale bar = 100 μm.

*Onion epidermal cell*: This study used onion inner epidermal cells as the model system to systematically investigate the fluorescence response characteristics under different environmental stresses after staining with 5 μM HJA‐MQ‐D for 5 min. The experiment involved treating the stained epidermal tissues with gradient concentrations of NaCl solution (0, 30, 50, 100 mM), Cd^2+^ solution (0, 30, 50, 70, 100 μM), 0.3 g/mL sucrose solution, H_2_O_2_ solution (0, 5, 10, 20, 50 mM) and air exposure conditions. The experimental results indicated that the fluorescence intensity of the cells in the NaCl treatment group increased with increasing concentration and treatment time (Figure 5a), but after more than 10 min, the probe fluorescence was quenched and plasmolysis phenomena appeared (Figure S19a). Similar fluorescence enhancement and quenching effects were observed in the sucrose treatment group (Figure S19b). In addition, both Cd^2+^ and H_2_O_2_ treatment groups exhibited an increase in fluorescence intensity (Figure 5b,c), and the fluorescence intensity increased with prolonged exposure time under air exposure conditions (Figure S19c). These results indicate that the HJA‐MQ‐D probe can effectively monitor changes in the cellular microenvironment.

**FIGURE 6:**
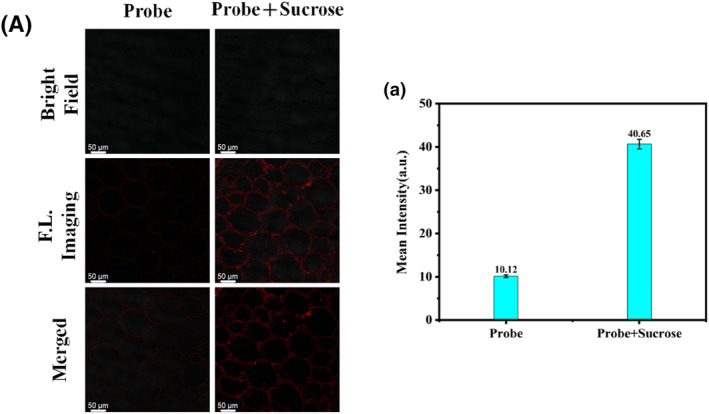
(A) Magnified views of cells from control and experimental groups. (a) Quantitative analysis of relative fluorescence intensity in the red channel for panel (A). Red channel: *λ*_ex_ = 638 nm, *λ*_em_ = 680–730 nm, scale bar = 50 μm.

*Mung bean slice cells*: The purchased mung bean seeds were sterilized in 75% ethanol for 30 s, rinsed three times with distilled water, and cultivated at 25°C for 7 days to obtain seedlings. The cultivated seedlings were sectioned, with control group sections treated with 5 μM of probe HJA‐MQ‐D stock solution for 5 min, while experimental group sections were first pretreated with 5 μM of probe HJA‐MQ‐D for 5 min followed by 0.3 g/mL sucrose solution for 5 min, allowing evaluation of the sucrose‐induced cellular viscosity changes. The experimental results indicated that pretreatment with 0.3 g/mL sucrose solution significantly increased intracellular viscosity in mung bean seedling sections compared to the control group (Figure 6) and (Figure S20), Quantitative analysis of the red channel fluorescence intensity revealed approximately 4‐fold, enhancement in relative fluorescence intensity in the treatment group.

*Mung bean roots*: To investigate the effect of salt stress on plant cell microviscosity, we performed imaging analysis of mung bean seedling root tips across a salt concentration gradient. Root tips from mung bean roots with consistent growth were selected and prepared into root tip sections. Slices from the experimental group were incubated with 5 μM probe HJA‐MQ‐D for 5 min, then transferred to buffered solutions containing 0 (control), 30, 50, or 100 mM NaCl for 30‐min treatment; control group slices were incubated simultaneously in salt‐free buffer. Confocal imaging results (Figure S21) revealed concentration‐dependent increases in red channel fluorescence signals from root tip cells as the salt concentration increased. These findings demonstrate that salt stress rapidly induces elevated intracellular viscosity with a concentration‐dependent effect, confirming probe HJA‐MQ‐D's utility for dynamically monitoring cellular microenvironment changes under salt stress.

*Imaging of whole plant*: To evaluate the applicability of the probe HJA‐MQ‐D for monitoring heavy metal stress and salt stress in plants, we established a dual‐model system using mung beans (*Vigna radiata* cv. Zhonglv No.1) and peanuts (*Arachis hypogaea* cv. Luhua 14) by applying exogenous heavy metal and salt stresses to simulate dynamic viscosity variations in plant tissues, with untreated groups serving as blank controls. In the experiment, mung bean and peanut seeds were sterilized with 75% alcohol for 30 s, rinsed three times with sterile water and cultured at room temperature in the dark. Firstly, mung bean and peanut seeds were cultured in distilled water for germination and then directly incubated under different concentrations of Cd^2+^ (0, 30, 50, 70, 100 μM) and NaCl (0, 100, 150, 200, 250 mM) stress conditions for different periods of time. After the culture was completed, all plant materials were uniformly immersed in 5 μM fluorescent probe HJA‐MQ‐D solution (dissolved in PBS buffer, pH 7.4) and incubated for 1 h in the dark, and the residual probe solution on the surface was washed off with water prior to imaging.

**FIGURE 7:**
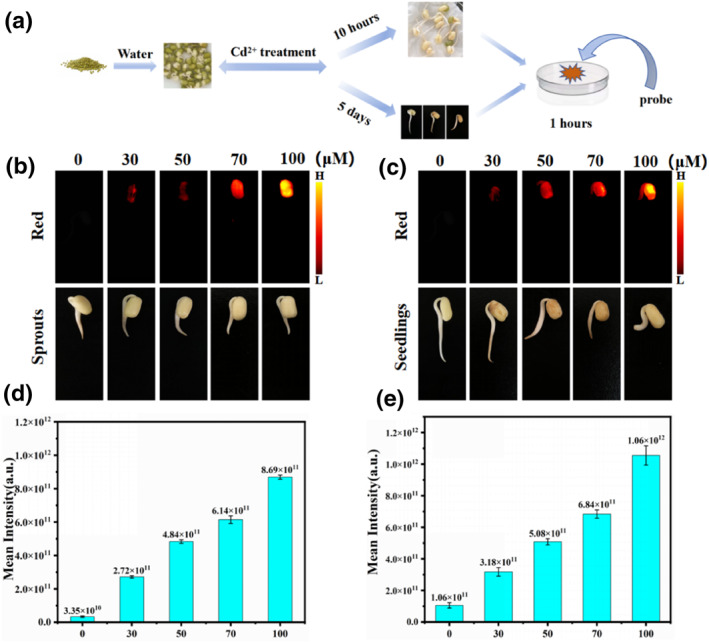
(a) Experimental procedure of heavy metal Cd^2+^ stress treatment on mung bean seeds. (b, c) Whole‐plant fluorescence imaging after incubation with different concentrations of Cd^2+^ (0, 30, 50, 70, 100 μM) for 10 h and 5 days, respectively. (d, e) Quantitative analysis of fluorescence intensity of whole plant imaging. *λ*_ex_ = 630 nm, *λ*_em_ = 699 nm.

**FIGURE 8:**
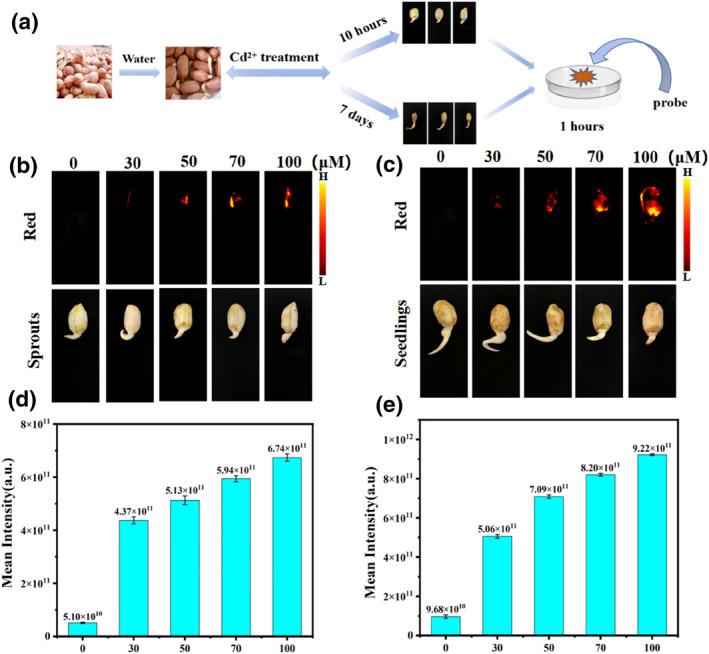
(a) Experimental procedure of heavy metal Cd^2+^ stress treatment on peanuts. (b, c) Whole‐plant fluorescence imaging after incubation with different concentrations of Cd^2+^ (0, 30, 50, 70, 100 μM) for 10 h and 7 days, respectively. (d, e) Quantitative analysis of fluorescence intensity of whole plant imaging. *λ*_ex_ = 630 nm, *λ*_em_ = 699 nm.

**FIGURE 9:**
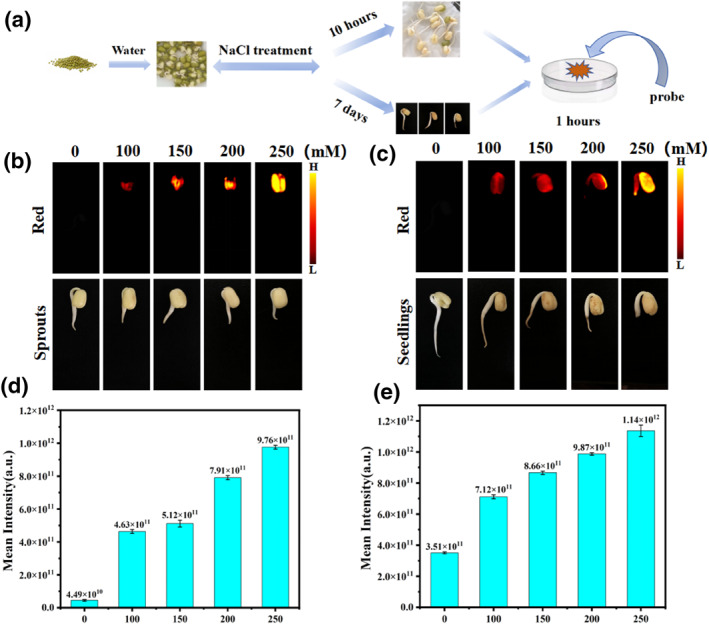
(a) Experimental procedure of heavy metal NaCl stress treatment on mung bean seeds. (b, c) Whole‐plant fluorescence imaging after incubation with different concentrations of NaCl (0, 100, 150, 200, 250 mM) for 10 h and 7 days, respectively. (d, e) Quantitative analysis of fluorescence intensity of whole plant imaging. *λ*_ex_ = 630 nm, *λ*_em_ = 699 nm.

**FIGURE 10:**
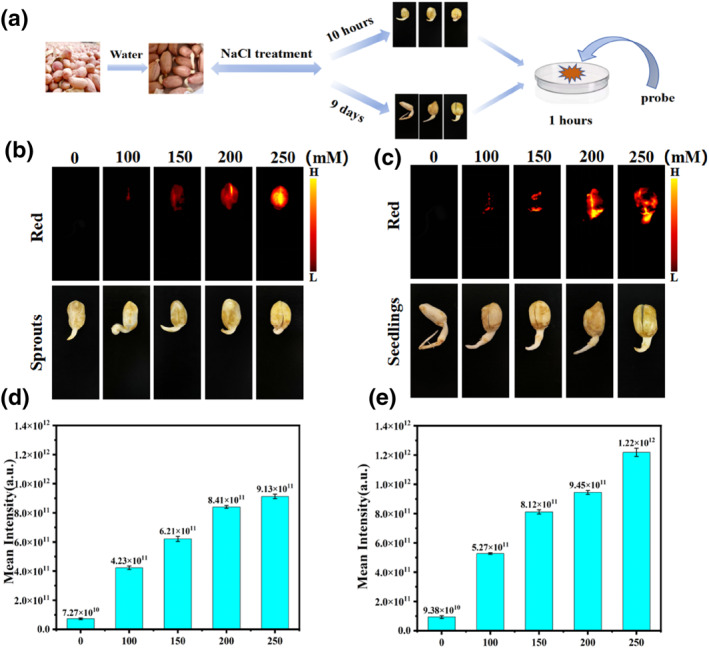
(a) Experimental procedure of heavy metal NaCl stress treatment on peanuts. (b, c) Whole‐plant fluorescence imaging after incubation with different concentrations of NaCl (0, 100, 150, 200, 250 mM) for 10 h and 9 days, respectively. (d, e) Quantitative analysis of fluorescence intensity of whole plant imaging. *λ*_ex_ = 630 nm, *λ*_em_ = 699 nm.

Figure 7b,c illustrates the variation in performance of mung bean after 10 h and 5 days of germination in different concentrations of Cd^2+^ (0, 30, 50, 70, 100 μM) solutions, whereas Figure 8b,c illustrates the growth of peanut after 10 h and 7 days of incubation at the same Cd^2+^ gradient. Whole‐plant fluorescence imaging results indicated (Figures 7d,e and 8d,e) that both plants exhibited a clear concentration‐ and time‐dependent response: the intensity of fluorescence signals in plant tissues was enhanced as the concentration of Cd^2+^ increased (30 → 100 μM) and the exposure time was prolonged (10 h → 5/7 days). This indicates that Cd^2+^ stress activates the probe response due to an increase in the viscosity level of the plant cells, and that the increase in fluorescence intensity is positively correlated with heavy metal stress and treatment duration. Figure 9b,c illustrates the changes in the performance of mung bean after 10 h and 7 days of germination in different concentrations of NaCl (0, 100, 150, 200, 250 mM) solutions, whereas Figure 10b,c shows the growth of peanut incubated under the same salt concentration gradient for 10 h and 9 days. Whole‐plant fluorescence imaging results indicate (Figures 9d,e and 10d,e) that both plants exhibited a significant concentration‐ and TD response: the fluorescence signal intensity of plant tissue indicated an enhancement with increasing salt concentration (100 → 250 mM) and prolonged exposure time (10 h → 7/9 days). This indicates that NaCl stress activates the probe response due to an increase in the intracellular viscosity level of the plant cells, and that the increase in fluorescence intensity is positively correlated with the salt stress intensity and treatment duration.

### 3.5 Viscosity detection in zebrafish

**FIGURE 11:**
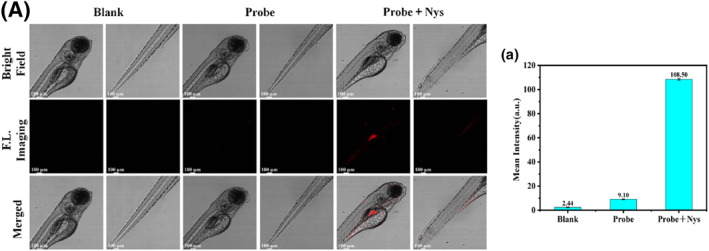
(A) Imaging of three groups of zebrafish. (a) Quantitative analysis of relative fluorescence intensity in the red channel for panel (A). Red channel: *λ*_ex_ = 638 nm, *λ*_em_ = 680–730 nm, scale bar = 100 μm.

Zebrafish are considered as an ideal model organism for fluorescence labeling research due to their unique advantages including transparent embryos for easy observation, high similarity to humans, and rapid organ development. However, since low cytotoxicity is a prerequisite for intracellular visualization, we assessed the biocompatibility of HJA‐MQ‐D using an MTT assay and examined its cytotoxicity at concentrations of 0, 5, 10, 15, and 20 μM prior to conducting zebrafish fluorescence imaging experiments. Results indicated that even after 24 h of treatment with 20 μM HJA‐MQ‐D, HeLa cell viability remained above 85% (Figure S22), indicating that HJA‐MQ‐D exhibits remarkably low toxicity and can be safely used for in vivo fluorescence imaging studies in zebrafish. Based on these characteristics, this study used 5‐day‐old zebrafish larvae as a model to verify the dynamic monitoring capability of the fluorescent probes for intracellular viscosity changes. The study established three experimental groups: the blank control group consisted of normally cultured 5‐day‐old zebrafish larvae; experimental group I used zebrafish incubated in 10 μM of fluorescent probe HJA‐MQ‐D solution for 30 min; and the experimental group II involved zebrafish first pretreated with 10 μM Nystatin (Nys) for 30 min and then transferred to 10 μM fluorescent probe HJA‐MQ‐D solution for another 30 min. All treatments were performed in culture medium at 28°C. The experimental results indicated that the blank control group (untreated) zebrafish larvae exhibited no significant fluorescence signal; experimental group I treated with only 10 μM fluorescent probe HJA‐MQ‐D exhibited weak fluorescence; while experimental group II pretreated with 10 μM Nys before probe loading exhibited significantly enhanced fluorescence signals. This gradient response (blank group < probe group < probe + Nys group) systematically verified the probe's capability to monitor cellular viscosity changes (Figure 11).

## 4 CONCLUSIONS

In conclusion, a novel water‐soluble NIR fluorescent probe HJA‐MQ‐D, was successfully synthesized using a facile and efficient route with excellent water solubility. The probe exhibited high sensitivity and selectivity due to the intramolecular TICT mechanism to achieve a specific response to viscosity changes. Validation experiments in a variety of biological model systems confirmed that HJA‐MQ‐D is capable of accurately monitoring the dynamic changes of viscosity in plant and animal cells, including plant cell models such as onion epidermal cells and mung bean seedling sections, as well as animal models such as zebrafish. Particularly, noteworthy is that the probe exhibited excellent performance for whole plant imaging. These research results fully demonstrate that HJA‐MQ‐D is a highly promising tool for cell viscosity visualization research, which provides a new technological tool for the evaluation of the microenvironment of biological systems.

## CONFLICT OF INTEREST STATEMENT

The authors declare no conflicts of interest.

## ETHICS STATEMENT

All animal protocols adhered to the care and usage guidelines established for laboratory animals by the Xuchang University. The experiments were sanctioned by the Xuchang University Medical Ethics Committee (Henan, China, ethics statement Reference No. 2025014).

## Supporting information

Supporting Information S1

## Data Availability

The data that supports the findings of this study are available in the supplementary material of this article.
