# Supplementary material for: A novel water‐soluble near‐infrared fluorescent probe for monitoring viscosity fluctuations in plants and zebrafish under abiotic stresses
Source: Smart Mol. 2026 Mar 19;4(2):e70043. doi: 10.1002/smo2.70043 (PMC13317596; doi:10.1002/smo2.70043)
Supplement: Supplementary file 1 — Supporting Information S1 [file SMO2-4-e70043-s001.docx]

**Supplementary information**

**A novel water-soluble near-infrared fluorescent probe for monitoring viscosity fluctuations in plants and zebrafish under abiotic stresses**

**Yixue Feng^a,b,1^, Keke Ding^b,1^, Yongxu Yang^b^, Quanliang Liu^c^, Silin Xu^c^, Chunhui Liu^b^, Zhihong Xu^a,b^*, Xufeng Hou^b,^*, Jihong Huang^d,*^, Tony D. James^e,f,*^**

*^a^ College of Chemistry, Zhengzhou University, Zhengzhou, 450052, PR China*

*^b^ Key Laboratory of Chemo/Biosensing and Detection of Xuchang, Key Laboratory of Micro-Nano Materials for Energy Storage and Conversion of Henan Province, Henan Joint International Research Laboratory of Nanomaterials for Energy and Catalysis, College of Chemical and Materials Engineering, Xuchang University, 461000, P.R. China*

*^c^ School of Science, Xuchang University, 461000, P.R. China*

*^d^ Collaborative Innovation Center of Functional Food by Green Manufacturing, Food and Pharmacy College, Xuchang University, Xuchang 461000, China*

*^e^ Department of Chemistry, University of Bath, Bath, BA2 7AY, UK*

*^f^ School of Chemistry and Chemical Engineering, Henan Normal University, Xinxiang 453007, China*

*KEYWORDS. Near infrared probe* • *viscosity sensing* • *large Stokes shift* • *biocompatibility* • *plant imaging*

**Correspondence**

**Zhihong Xu, Xufeng Hou, Jihong Huang and Tony D. James.**

**Email:** [xuzhihong1980@xcu.edu.cn](mailto:xuzhihong1980@xcu.edu.cn), [hxfst1979@163.com](mailto:hxfst1979@163.com), [huangjih1216@126.com](mailto:huangjih1216@126.com) and [t.d.james@bath.ac.uk](mailto:t.d.james@bath.ac.uk) (Tony D. James)

1. **Materials and Instrumentation**

^1^H NMR and ^13^C NMR spectra were recorded on a Bruker AV400 NMR spectrometer in DMSO-d_6_ or CDCl_3_ solutions or Methanol-d4. UV spectra were recorded on a UV-2550 spectrophotometer. Fluorescence spectra were measured using a FL-7100 fluorescence spectrophotometer (EX slit: 10.0 nm, EM Slit: 10.0 nm, Voltage: 700 V). High-resolution mass spectra were measured using a UPLC G2-XS Qtof from Waters. The fluorescence images were obtained with a Leica DMI 8 laser scanning confocal microscope. The values of pH were recorded on a Lichen Technology LC - PH - 3LT Desktop pH Meter. Whole plants were imaged using the Tanon ABL X6 imaging system (λ_ex_＝630 nm, λ_em_＝699 nm).

*m-Aminophenol* (99%), 1-Bromo-3-chloropropane (99%), Phosphoryl Chloride (99%), 4-Methylquinoline (98%), diethylene glycol 2-bromoethyl methyl ether (98%), Sodium Carbonate (99.9%), *N,N*-Dimethylformamide, Acetonitrile, Ethanol, Methanol, Dichloromethane are purchased from Shanghai Titan Technology Co., Ltd. and were not purified before use.

1. **Key Performance Comparison of Similar Probes**

**Table S1.** Representative Near-Infrared/Plant-Compatible Viscosity Probe Comparison

|  | **λ_em_** | **Stokes Shift** | **pH** | **Mechanism** | **Inhibition of Plant Autofluorescence** | **Bio-compatibility** | **Whole-plant** | **Root Imaging** | **Bio-imaging** | **Ref.** |
| --- | --- | --- | --- | --- | --- | --- | --- | --- | --- | --- |
|  | 710 nm | 30 nm | 6-9 | TICT | Good | Moderate | No | Yes | HepG2 cells/ Zebrafish/ Arabidopsis root | 1 |
|  | 723 nm | 78 nm | 4-10 | TICT | Good | Moderate | No | No | HeLa cells/Mice | 2 |
|  | 675 nm | 85 nm | 2-11 | TICT | Moderate | Moderate | No | No | HeLa cells/Mice | 3 |
|  | 720 nm | 40 nm | 6-10 | TICT | Good | Good | No | Yes | MCF-7cells Zebrafish/ /Rice roots | 4 |
|  | 588 nm | 73 nm | 4-10 | TICT | Moderate | Moderate | No | No | HeLa cells/Mice | 5 |
|  | 590 nm | 130 nm | 4-9 | TICT | Moderate | Moderate | No | No | HeLa cells/ Zebrafish | 6 |
|  | 688 nm | 118 nm | 5-9 | TICT | Good | Moderate | No | No | HeLa cells | 7 |
|  | 605 nm | 125 nm | 6-9 | TICT | Moderate | Good | No | No | HepG2 cells/ Zebrafish/ Onion epidermal cells | 8 |
|  | 560 nm | 160 nm | 5-10 | TICT | Moderate | Moderate | No | No | HeLa cells/Mung bean sprouts | 9 |
|  | 579 nm | 34 nm | --- | TICT | Moderate | Moderate | No | No | HeLa cells | 10 |
|  | 650 nm | 80 nm | 8-12 | TICT | Moderate | Moderate | No | No | HepG2 cells/ Zebrafish | 11 |
|  | 670 nm | 190 nm | 4-10 | AIE | Moderate | Moderate | No | No | A549 cells/Mice | 12 |
|  | 650 nm | 145 nm | 3-8 | AIE | Moderate | Moderate | No | No | HeLa cells | 13 |
|  | 674 nm | 227 nm | 4-10 | AIE | Moderate | Moderate | No | No | HUVEC cells/MCF-7 cells/Mice | 14 |
|  | 610 nm | 260 nm | --- | ESIPT  TICT | Moderate | Moderate | No | No | HepG2 cells | 15 |
|  | 698 nm | 88 nm | 2-8 | TICT | Good | Good | Yes | No | Onion epidermal cells/ Mung bean cell slices/ Mung bean root / Whole mung beans and peanuts/ Zebrafish | This  Work |

**Table S2.** Comparison of Key Performance Parameters for Probes with Similar Structures

|  | **λex/λem** | **Stokes Shift** | **Mechanism** | **Inhibition of Plant Autofluorescence** | **Water-**  **soluble** | **Bioimaging** | **Ref.** |
| --- | --- | --- | --- | --- | --- | --- | --- |
|  | 492/514 | 22 nm | ICT | Moderate | ~ | Test strip detection | 16 |
|  | 560/613 | 53 nm | TICT | Moderate | ~ | MDA-MB-231 cell/MCF- 10A cell/mice | 17 |
|  | 445/500 | 55 nm | ICT | Moderate | ~ | Sensing gels and labels | 18 |
|  | 470/560 | 90 nm | TICT | Moderate | ~ | H9c2 cells/zebrafish | 19 |
|  | 480/560 | 120 nm | TICT  ESIPT | Moderate | ~ | C. elegans | 20 |
|  | 445/568 | 123 nm | ICT | Moderate | ~ | Test strips | 21 |
|  | 520/652 | 132 nm | TICT | Moderate | ~ | Nthy-ori3-1 cells/TPC-1 cells/mice | 22 |
|  | 600/735 | 135 nm | TICT | Good | Moderate | A549 cell | 23 |
|  | 510/675 | 165 nm | TICT | Moderate | ~ | HeLa/MCF-7 cells/Zebrafish | 24 |
|  | 369/491 | 122 nm | ICT  ESIPT | Moderate | ~ | Danio rerio Gill cells/Zebrafish | 25 |
|  | 867/907 | 40 nm | ~ | Good | ~ | Huh-7 cell/Mice | 26 |
|  | 610/698 | 88 nm | TICT | Good | Good | Onion epidermal cells/ Mung bean cell slices/ Mung bean root /Whole mung beans and peanuts/ Zebrafish | This work |

1. **DFT calculations**

Firstly, a gjf file for **HJA-MQ-D** was created using GaussView 5.0. Using Gaussian 16 software, geometric structure optimization was performed on both planar and 90 degree twisted configurations at the PBE0/6-311G (d, p) level. Based on the optimized stable structure, the energy difference and excited state properties between different configurations, including fluorescence oscillator strength (fem), were calculated using TD-DFT method.

1. **Synthesis**

A mixture of m-aminophenol (0.213 g, 1.95 mmol), 1-bromo-3-chloropropane (0.406 g, 0.98 mmol), and sodium carbonate (0.309 g) in DMF (4 mL) was stirred under reflux for 12 h, affording **Compound 2** (0.294 g, 70% yield). ^1^H NMR (400 MHz, Chloroform-*d*, Figure S1) δ 6.66 (d, *J* = 8.0 Hz, 1H), 6.06 (d, *J* = 8.0 Hz, 1H), 4.61 (d, *J* = 5.7 Hz, 1H), 3.09 (q, *J* = 5.1 Hz, 4H), 2.67 (dd, *J* = 14.3, 7.3 Hz, 4H), 1.98 (ddt, *J* = 15.5, 6.6, 4.6 Hz, 4H). ^13^C NMR (100 MHz, Chloroform-*d*, Figure S2) δ 151.74, 143.94, 126.73, 114.31, 107.81, 102.91, 50.21, 49.54, 27.21, 22.38, 21.62, 20.96.

Compound 2 (0.359 g, 2.17 mmol) was reacted with POCl₃ (0.5 mL, 5.43 mmol) to give **Compound 3**^27^ (0.419 g, 89% yield). ^1^H NMR (400 MHz, DMSO-*d*_6_, Figure S3) δ 11.80 (s, 1H), 9.29 (s, 1H), 6.90 (s, 1H), 3.20 (q, *J* = 5.7 Hz, 4H), 2.54 (t, *J* = 6.3 Hz, 2H), 2.47 (q, *J* = 5.1, 3.8 Hz, 2H), 1.76 (q, *J* = 5.9 Hz, 4H). ^13^C NMR (100 MHz, DMSO-*d*_6_, Figure S4) δ 192.08, 158.87, 149.68, 131.39, 113.84, 110.47, 104.64, 49.99, 49.54, 26.99, 21.50, 20.41, 19.73.

4-Methylquinoline (0.8 mL, 5.75 mmol) was treated with diethylene glycol 2-bromoethyl methyl ether (4.95 mL, 17.25 mmol) to furnish **Compound 5**^28^ (1.523 g, 70% yield). ^1^H NMR (400 MHz, DMSO-*d*_6_, Figure S5) δ 9.48 (d, *J* = 6.1 Hz, 1H), 8.81 (d, *J* = 9.0 Hz, 1H), 8.68 (dd, *J* = 8.5, 1.4 Hz, 1H), 8.38 (ddd, *J* = 8.7, 6.9, 1.4 Hz, 1H), 8.21 (dd, *J* = 11.1, 6.9 Hz, 2H), 5.40 (t, *J* = 4.9 Hz, 2H), 4.09 (t, *J* = 4.9 Hz, 2H), 3.64 (dd, *J* = 3.9, 2.0 Hz, 2H), 3.50 (s, 2H), 3.44 – 3.42 (m, 2H), 3.42 – 3.40 (m, 2H), 3.15 (s, 3H). ^13^C NMR (100 MHz, DMSO-*d*_6_, Figure S6) δ 159.34, 149.65, 137.39, 135.44, 130.03, 129.30, 127.57, 122.72, 120.02, 71.61, 70.31, 70.05, 69.96, 68.23, 58.51, 56.92, 20.26. The synthetic route is shown in Scheme S1.


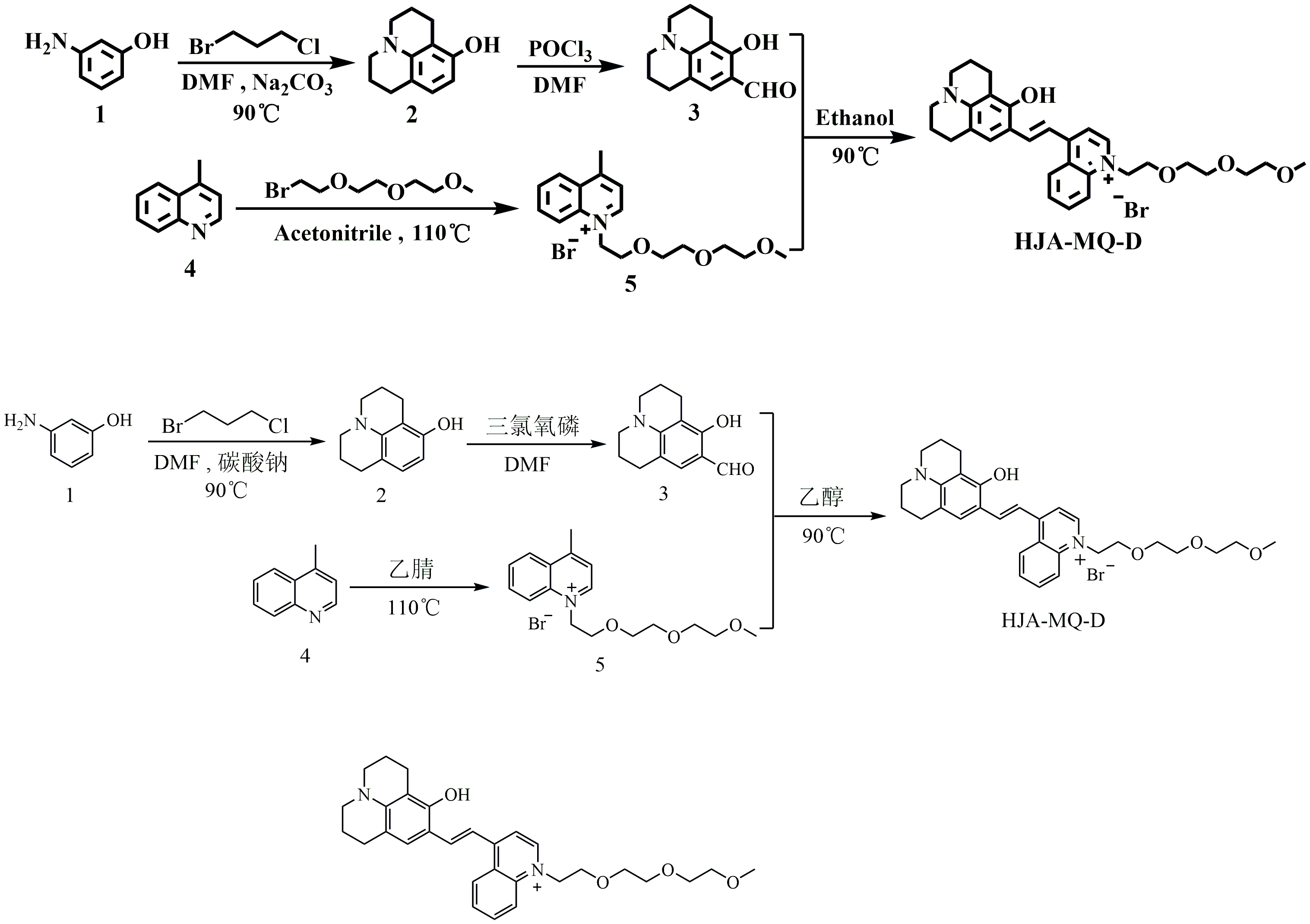


**Scheme S1.** Syntheticroute for the preparation of **HJA-MQ-D**

Compound 1, 2, and 3 were synthesized according to the synthetic route described above. Compound 1 (136.28 mg, 0.628 mmol) and compound 2 (179.06 mg, 0.628 mmol) were dissolved in anhydrous ethanol (8 mL). The mixture was the refluxed at 90 ℃ for 12 h. Upon cooling to room temperature, the crude product was concentrated under reduced pressure and subsequently purified by preparative thin-layer chromatography (TLC) with a mixed solvent system of dichloromethane/methanol (40:1 v/v) as eluent. The final product **HJA-MQ-M** was obtained and collected as a deep blue solid (137.2 mg, 45% yield). ^1^H NMR (600 MHz, DMSO-*d*_6_, Figure S10) δ 8.87 (d, *J* = 8.4 Hz, 1H), 8.81 (d, *J* = 6.8 Hz, 1H), 8.39 (d, *J* = 15.1 Hz, 1H), 8.20 (d, *J* = 8.8 Hz, 1H), 8.11 (t, *J* = 7.8 Hz, 1H), 8.03 (d, *J* = 6.8 Hz, 1H), 7.87 (t, *J* = 7.7 Hz, 1H), 7.76 (d, *J* = 15.1 Hz, 1H), 7.61 (s, 1H), 4.31 (s, 3H), 3.30 (t, *J* = 5.8 Hz, 2H), 3.26 (t, *J* = 5.7 Hz, 2H), 2.70 (t, *J* = 6.3 Hz, 2H), 2.61 (t, *J* = 6.4 Hz, 2H), 1.87 (dp, *J* = 12.1, 6.2 Hz, 4H). ^13^C NMR (151 MHz, DMSO-*d*_6_, Figure S11) δ 155.24, 153.44, 148.24, 145.95, 140.34, 139.22, 134.64, 128.34, 126.41, 126.38, 125.71, 119.08, 115.84, 112.35, 112.31, 110.16, 107.20, 50.13, 49.42, 43.86, 27.25, 21.80, 21.39, 20.90.

Compound 1 (124.12 mg, 0.572 mmol) and compound 3 (171.14 mg, 0.572 mmol) were dissolved in anhydrous ethanol (8 mL). The mixture was the refluxed at 90 ℃ for 12 h. Upon cooling to room temperature, the crude product was concentrated under reduced pressure and subsequently purified by preparative thin-layer chromatography (TLC) with a mixed solvent system of dichloromethane/methanol (30:1 v/v) as eluent. The final product **HJA-MQ-E** was obtained and collected as a deep blue solid (112.03 mg, 39.3 % yield). ^1^H NMR (400 MHz, DMSO-*d*_6_, Figure S12) δ 8.90 (d, *J* = 7.5 Hz, 2H), 8.43 (d, *J* = 15.1 Hz, 1H), 8.32 (d, *J* = 8.8 Hz, 1H), 8.15 – 8.05 (m, 2H), 7.90 – 7.75 (m, 2H), 7.65 (s, 1H), 4.80 (d, *J* = 7.2 Hz, 2H), 3.33 – 3.25 (m, 4H), 2.67 (dt, *J* = 34.8, 6.4 Hz, 4H), 1.88 (dd, *J* = 9.2, 5.2 Hz, 4H), 1.52 (t, *J* = 7.1 Hz, 3H). ^13^C NMR (100 MHz, DMSO-*d*_6_, Figure S13) δ 155.34, 153.55, 148.33, 145.02, 140.60, 138.14, 134.75, 128.19, 126.85, 126.45, 126.07, 118.84, 115.87, 112.75, 112.39, 110.24, 107.22, 51.09, 50.15, 49.46, 27.27, 21.83, 21.39, 20.93, 15.40. The synthetic route is shown in Scheme S2.

**Scheme S2.** Synthetic route for the preparation of **HJA-MQ-M** and **HJA-MQ-E**


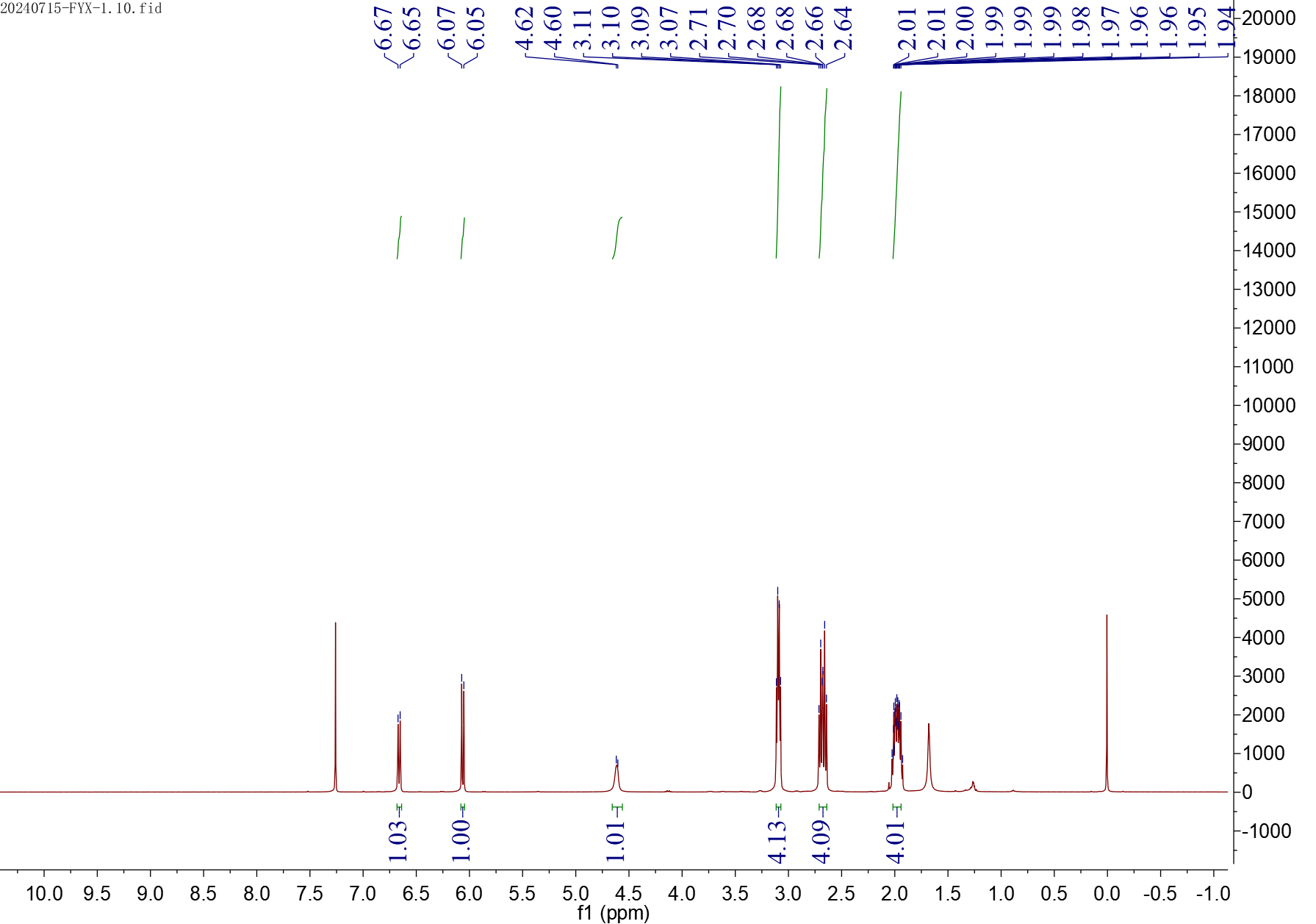


**Figure S1.** ^1^H NMR spectrum of **Compound 2.**

**
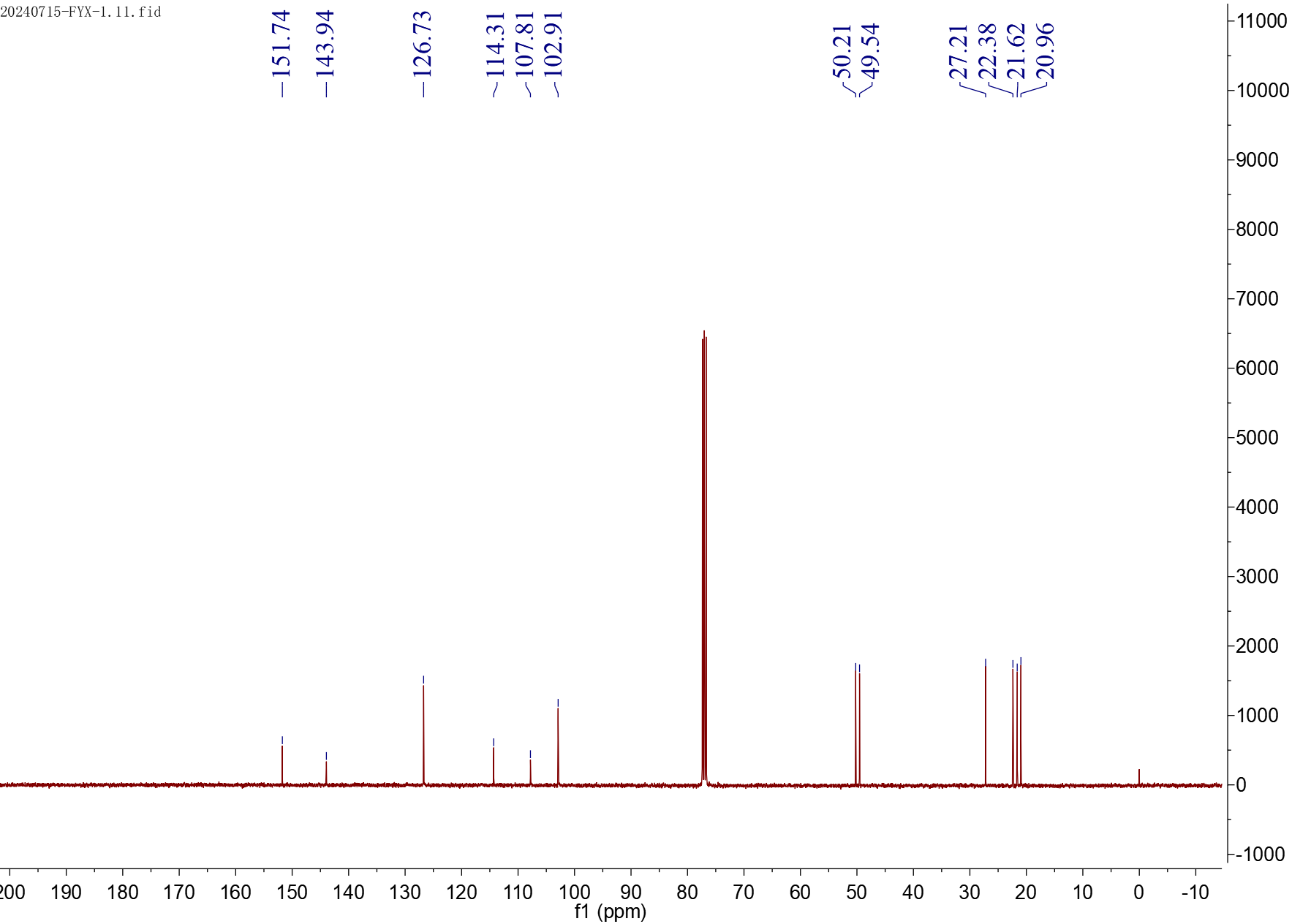
**

**Figure S2.** ^13^C NMR spectrum of **Compound 2.**


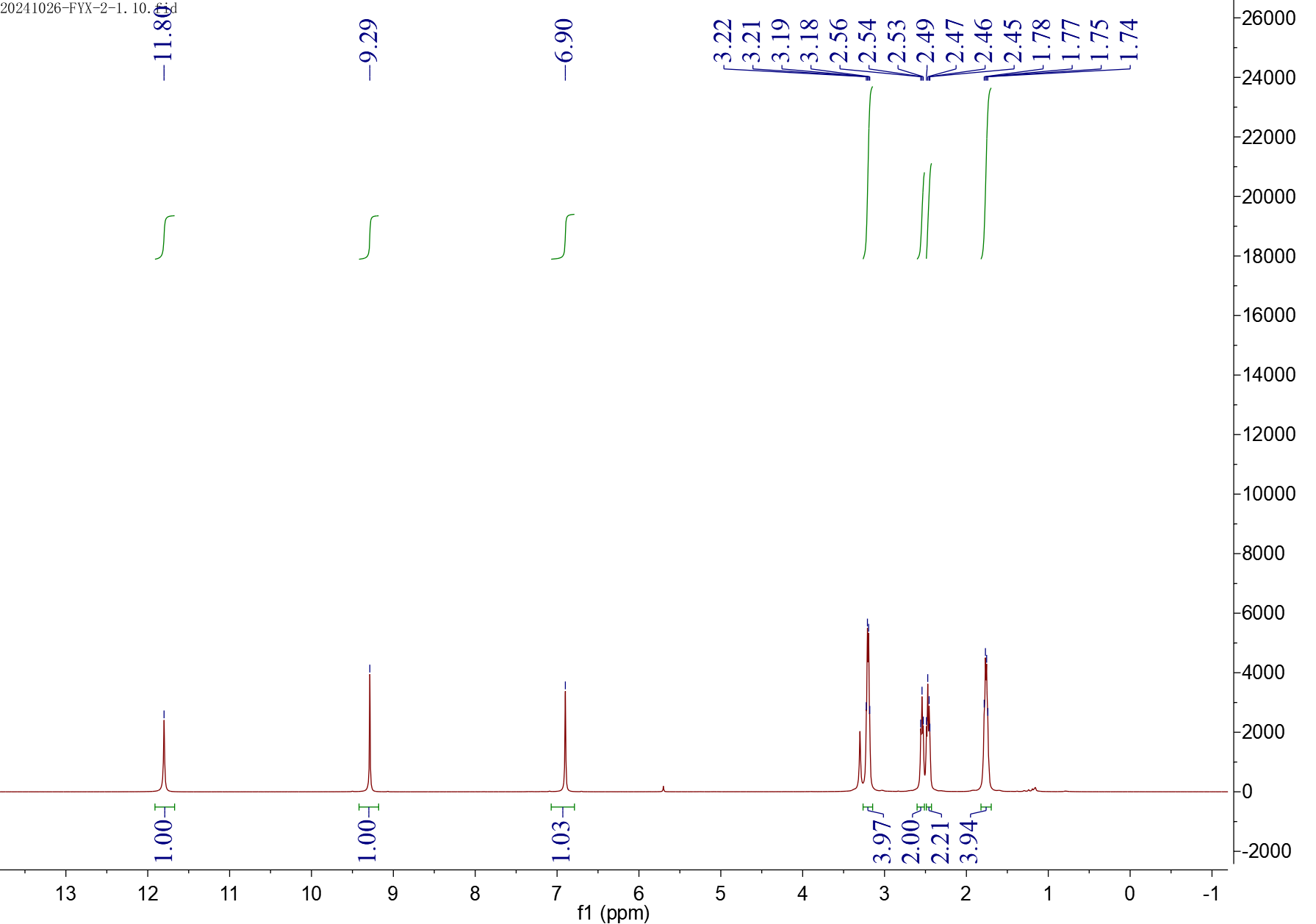


**Figure S3.** ^1^H NMR spectrum of **Compound 3.**

**
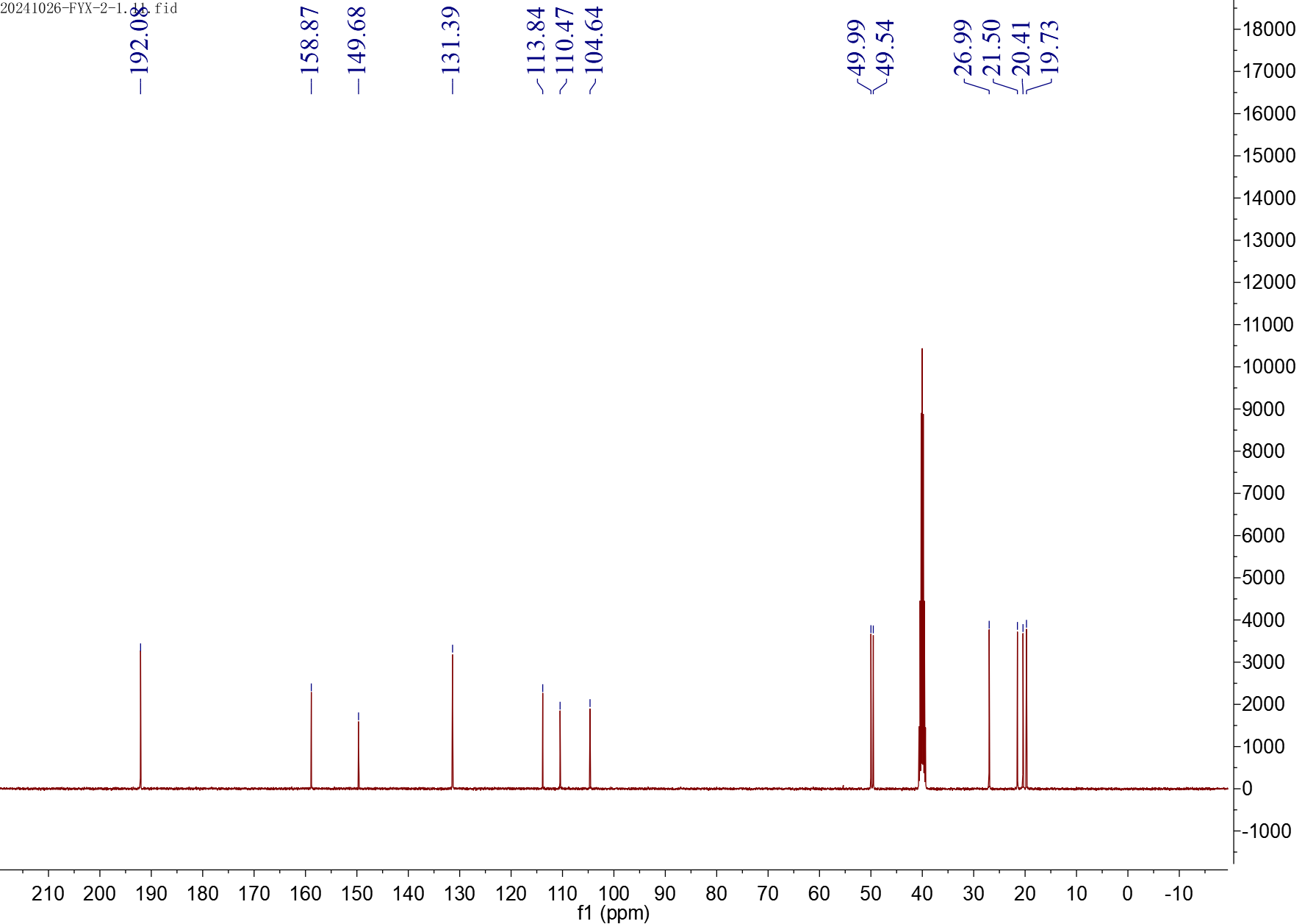
**

**Figure S4.** ^13^C NMR spectrum of **Compound 3.**

**Figure S5.** ^1^H NMR spectrum of **Compound 5.**

**
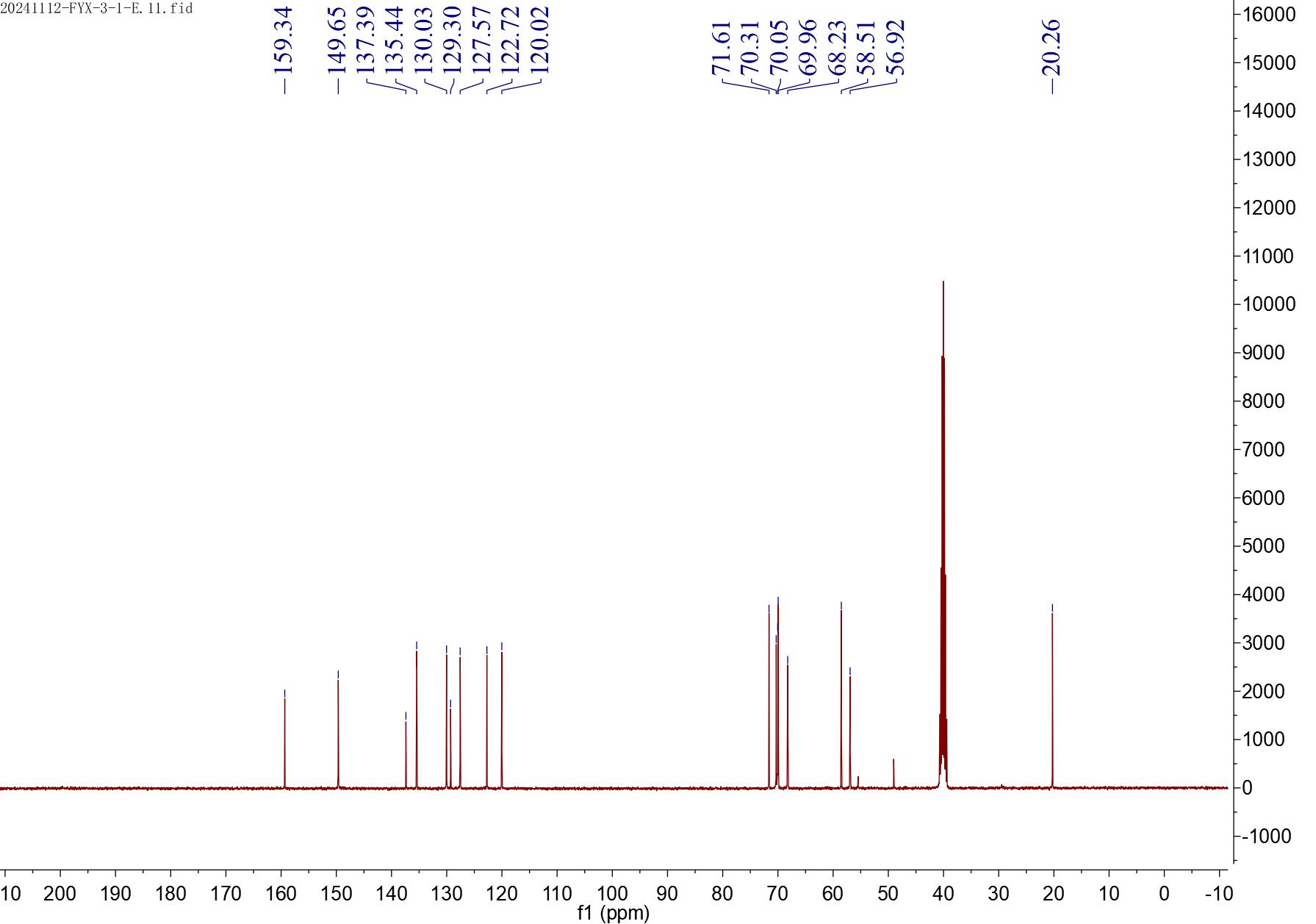
**

**Figure S6.** ^13^C NMR spectrum of **Compound 5.**


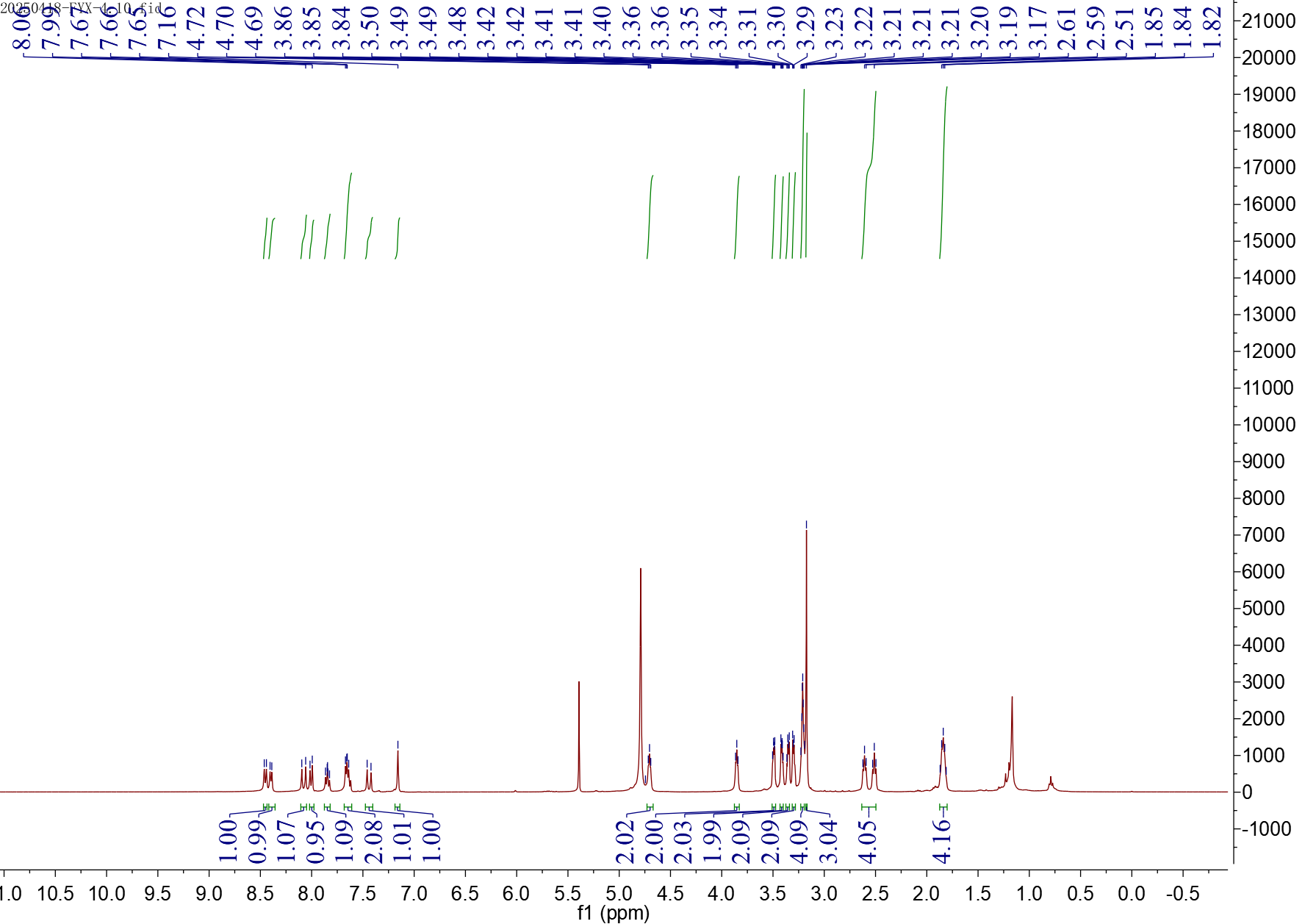


**Figure S7.** ^1^H NMR spectrum of **HJA-MQ-D.**


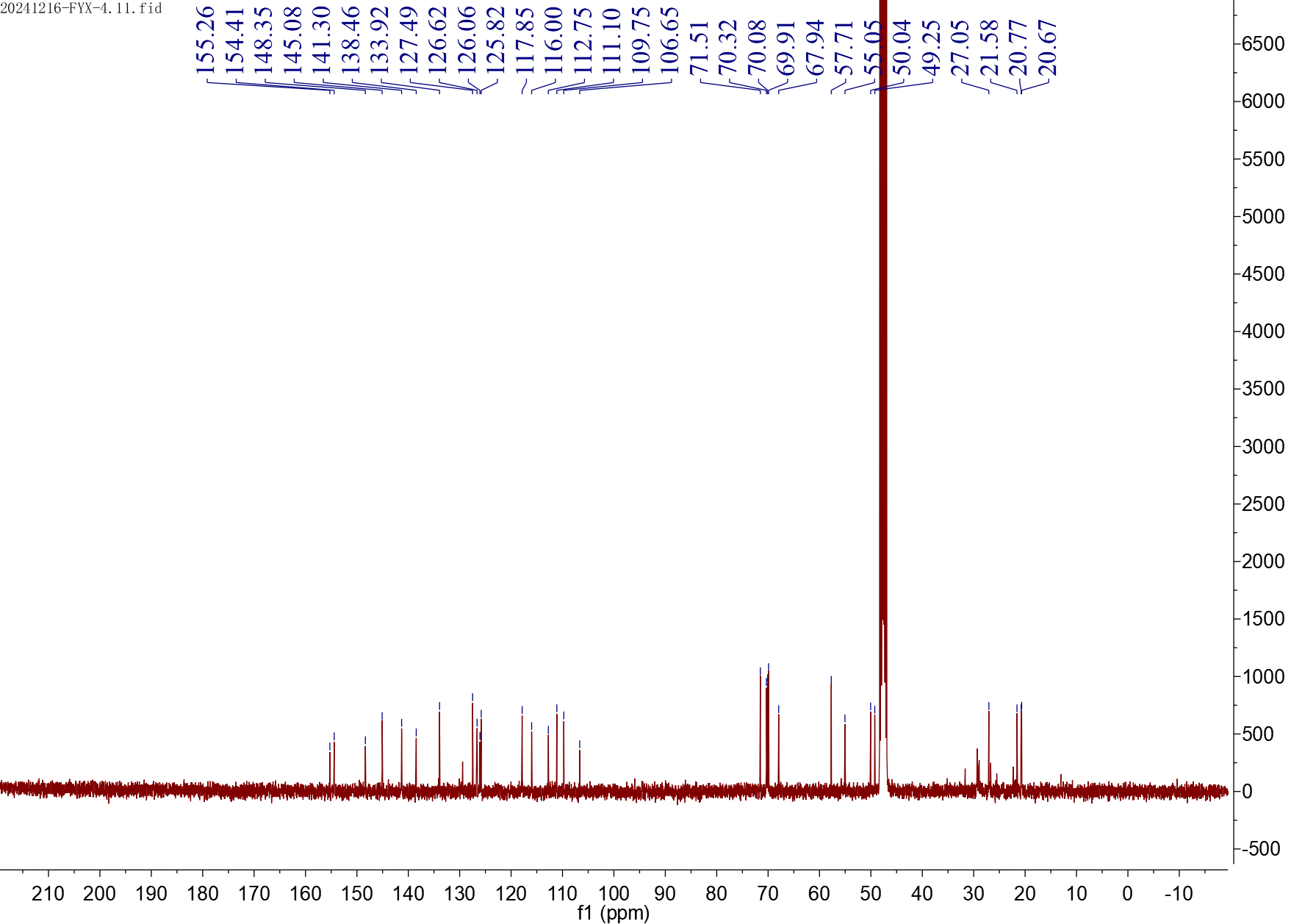


**Figure S8.** ^13^C NMR spectrum of **HJA-MQ-D.**


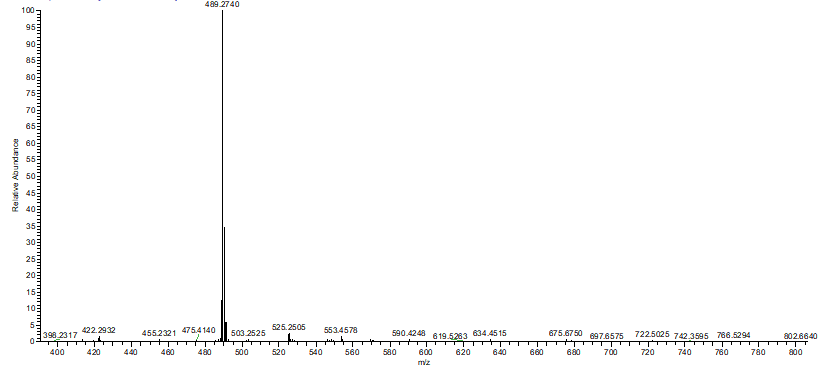


**Figure S9.** ESI-MS spectrum of the probe **HJA-MQ-D**.

**
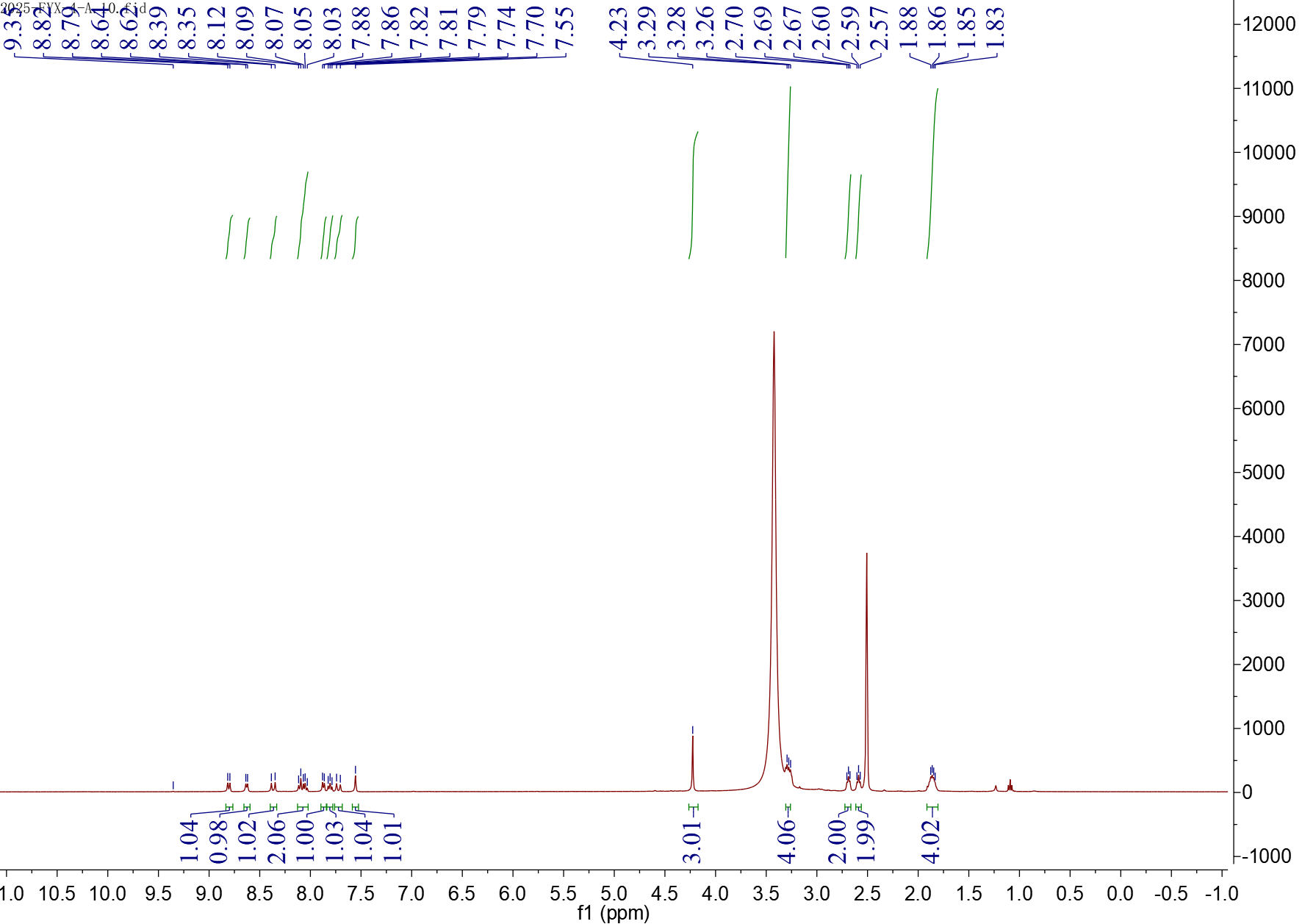
**

**Figure S10.** ^1^H NMR spectrum of **HJA-MQ-M.**

**Figure S11.** ^13^C NMR spectrum of **HJA-MQ-M.**


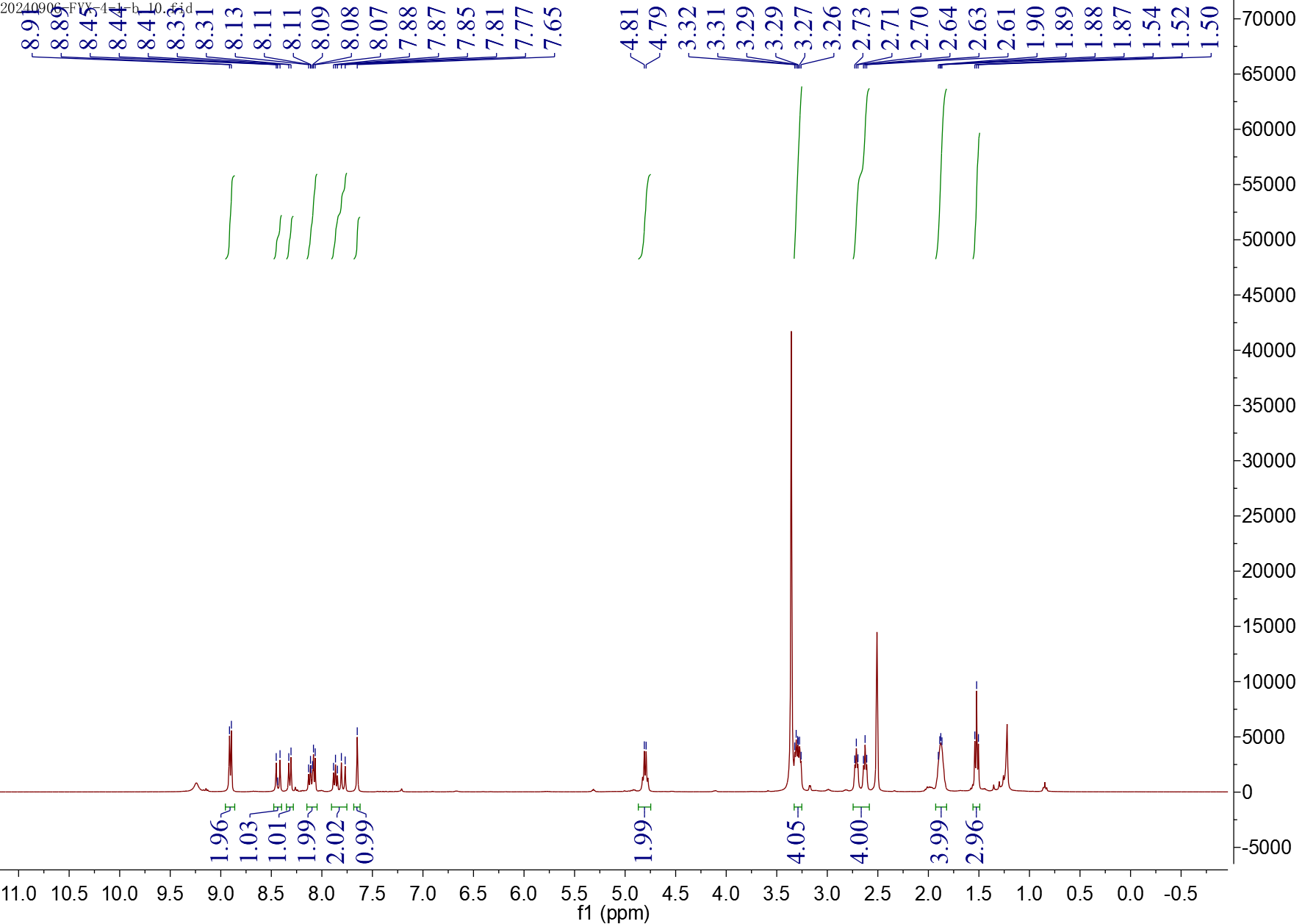


**Figure S12.** ^1^H NMR spectrum of **HJA-MQ-E.**

**
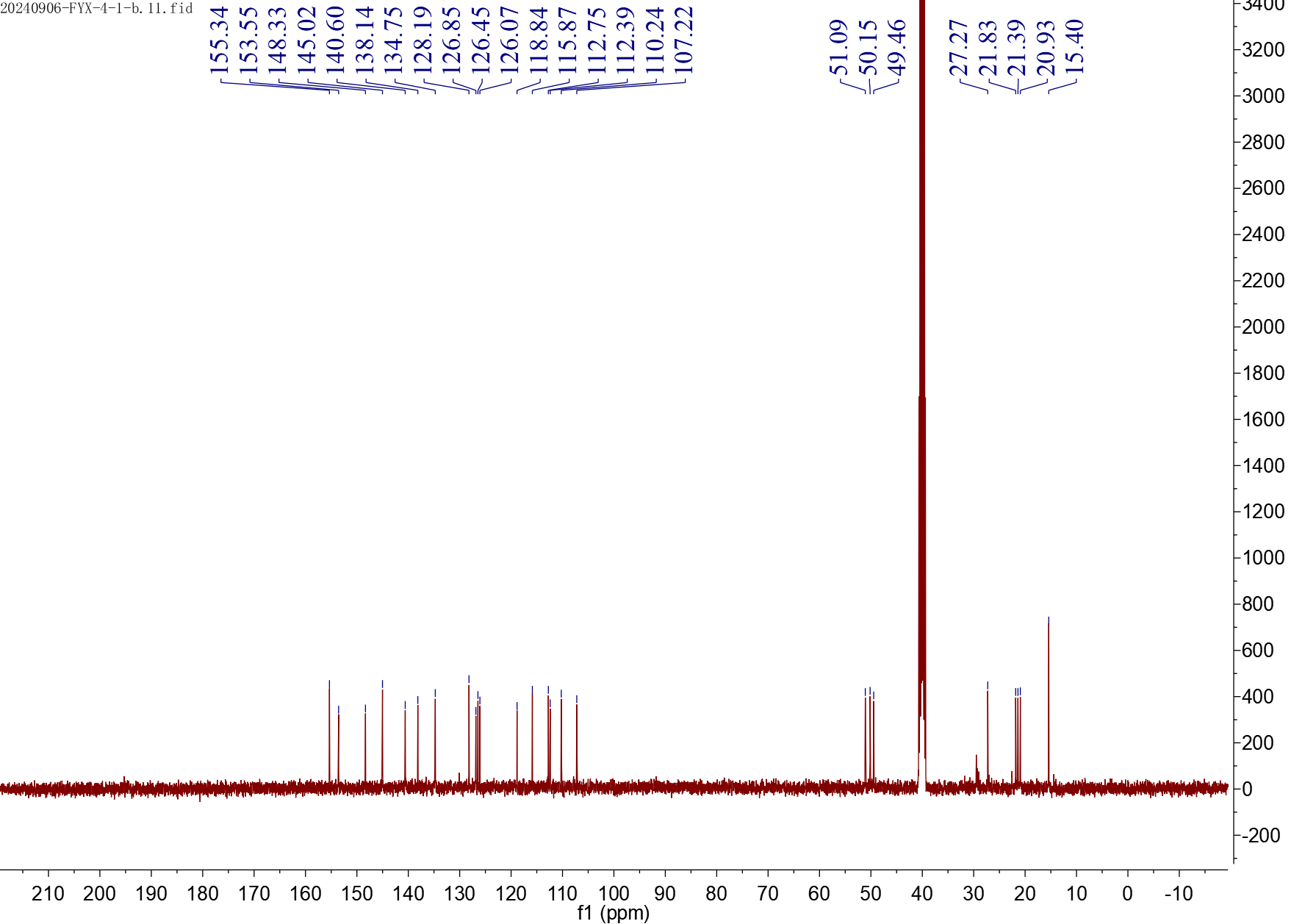
**

**Figure S13.** ^13^C NMR spectrum of **HJA-MQ-E.**

1. **Spectroscopic Analysis**

Figure S14 displays the UV-Vis absorption spectral characteristics of the probe **HJA-MQ-D**. Meanwhile, we systematically investigated the spectral response characteristics of the probe to 27 potential interfering substances (1. Zn^2+^, 2. Ni^2+^, 3. K^+^, 4. Co^2+^, 5. Ba^2+^, 6. Mn^2+^, 7. Fe^3+^, 8. Cu^2+^, 9. Cd^2+^, 10. Na^+^, 11. Ca^2+^, 12. N_2_H_4_ , 13. S^2-^, 14. F^-^, 15. Cl^-^, 16. Br^-^, 17. ClO^-^, 18. HSO_3_^-^, 19. Proline, 20. Glutamic acid, 21. Lysine, 22. Arginine, 23. Tryptophan, 24. Phenylalanine, 25. Cysteine, 26. Glutathione, 27. Homocysteine, 28. Blank) in a 50% glycerol-PBS system. The experimental results indicated that the probe exhibited excellent anti-interference ability in a 50% glycerol-PBS system (Figure S15).


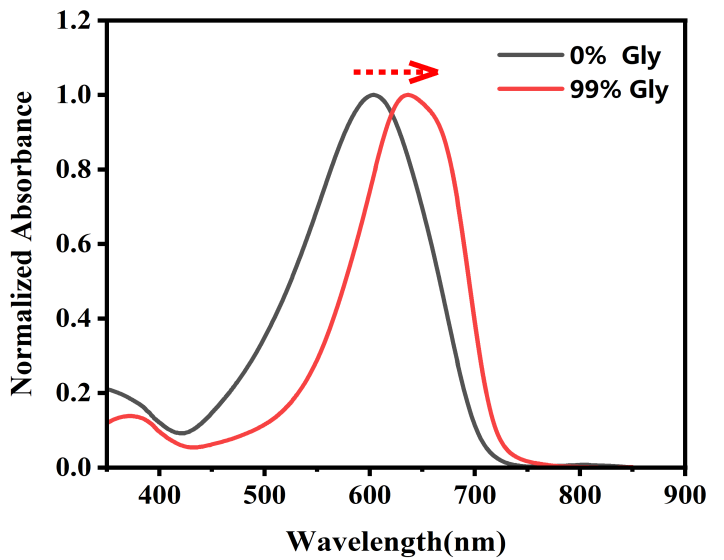


**Figure S14.** UV-vis absorption spectra of probe **HJA-MQ-D** (10 μM) in pure PBS (0% glycerol) and 99% glycerol solution.


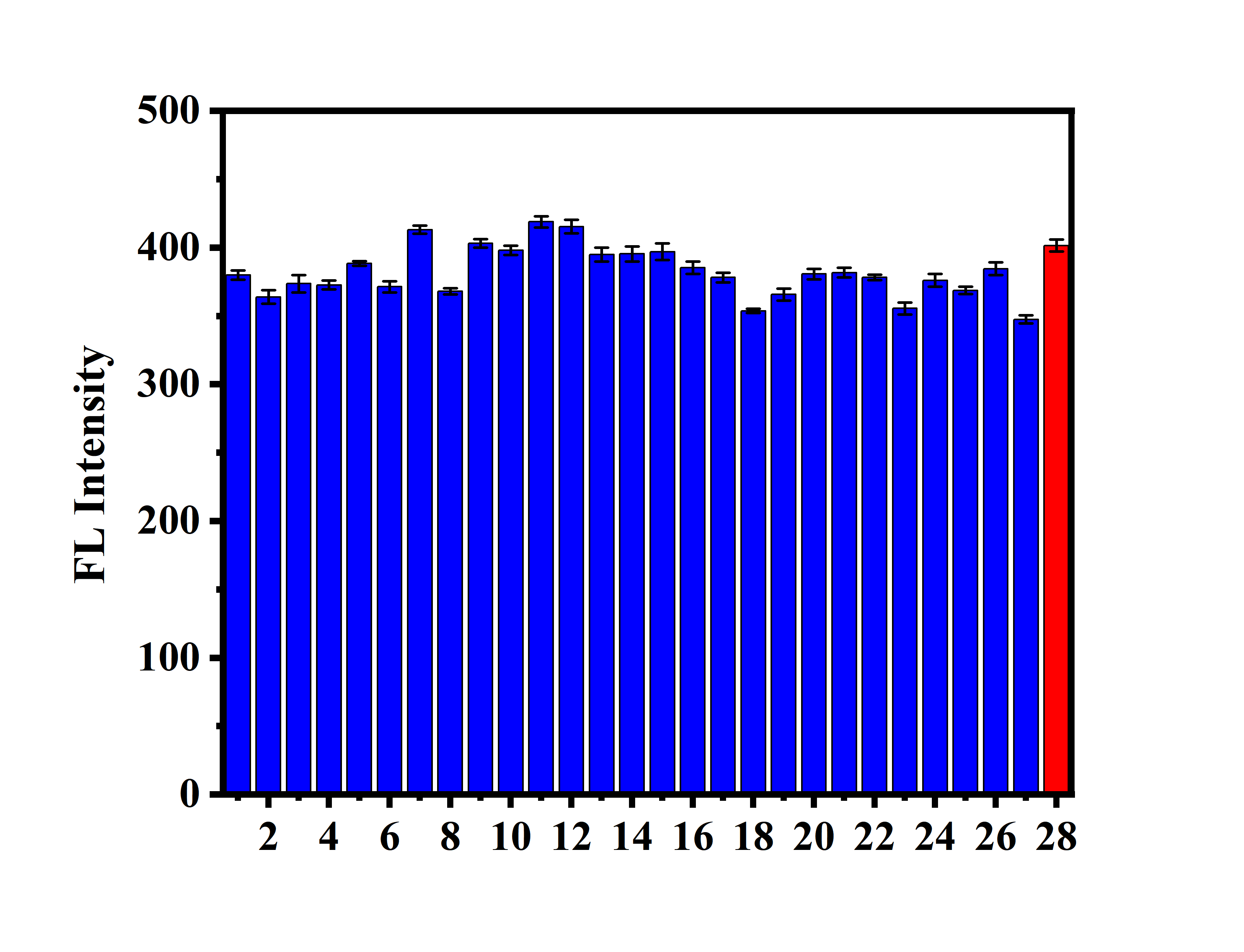


**Figure S15.** Fluorescence spectra of probe **HJA-MQ-D** (10 μM) in 50% glycerol-PBS mixture when they coexist with other different kinds of 20 μM analytes (1. Zn^2+^, 2. Ni^2+^, 3. K^+^, 4. Co^2+^, 5. Ba^2+^, 6. Mn^2+^, 7. Fe^3+^, 8. Cu^2+^, 9. Cd^2+^, 10. Na^+^, 11. Ca^2+^, 12. N_2_H_4_ , 13. S^2-^, 14. F^-^, 15. Cl^-^, 16. Br^-^, 17. ClO^-^, 18. HSO_3_^-^, 19. Proline, 20. Glutamic acid, 21. Lysine, 22. Arginine, 23. Tryptophan, 24. Phenylalanine, 25. Cysteine, 26. Glutathione, 27. Homocysteine, 28. Blank). The excitation wavelength was 610 nm. Slits = 10/10 nm.

1. **Comparison of Water-Soluble Quantitative Data**

We have supplemented the main text's water-solubility comparison section with a detailed quantitative data table (Table S3). This table systematically lists the measured fluorescence intensities of the three probes (**HJA-MQ-M**, **HJA-MQ-E**, and **HJA-MQ-D**) across a concentration range of 50 to 750 μM. These quantitative data clearly reveal the following key conclusions: The tabulated data show that the fluorescence intensities of **HJA-MQ-M** and **HJA-MQ-E** plateau after 350 μM and 250 μM, respectively, indicating near-saturated dissolution. In contrast, the fluorescence intensity of **HJA-MQ-D** continues to increase significantly throughout the tested concentration range (up to 450 μM), quantitatively demonstrating its higher apparent solubility.

**Table S3.** Comparison Table of Water-Soluble Quantitative Data for Three Types of Probes

|  | 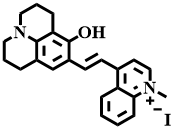 | 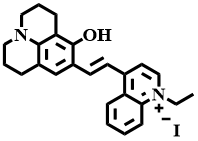 | 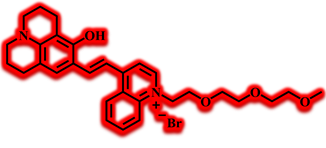 |
| --- | --- | --- | --- |
| **μM** | **FL Intensity** | **FL Intensity** | **FL Intensity** |
| **50** | 61.23 | 38.86 | 83.62 |
| **100** | 92.2 | 61.8 | 118.1 |
| **150** | 123.8 | 89.77 | 159.3 |
| **200** | 151.8 | 124.2 | 188.2 |
| **250** | 180.3 | 128.9 | 221.5 |
| **300** | 185.9 | 123.5 | 255.2 |
| **350** | 186.7 | 121.3 | 286.4 |
| **400** | 180.2 | 118.2 | 310.4 |
| **450** | 164.9 | 114.5 | 315.2 |
| **500** | 157.3 | 111.3 | 310.5 |
| **550** | 149.9 | 107.7 | 292.4 |
| **600** | 140 | 101.1 | 285.6 |
| **650** | 130.5 | 95.6 | 278.9 |
| **700** | 121.6 | 91.29 | 269.1 |
| **750** | 118.2 | 83.8 | 258.1 |

1. **Complex Biological Environment Simulation and Probe Response Specificity Validation**

Through orthogonal control experiments simulating multiple complex biological environments, we confirmed that the probe's fluorescence activation is primarily driven by viscosity (Figure S16), with minimal interference from polarity, protein binding, and other factors. Multispectral and competitive experiments collectively confirm that although the probe exhibits weak interactions with proteins (Table S4, Figure S17), this binding does not compromise its specific response to viscosity (Figure S18). Under simulated physiological viscosity-changing conditions, the probe accurately reflects viscosity variations.


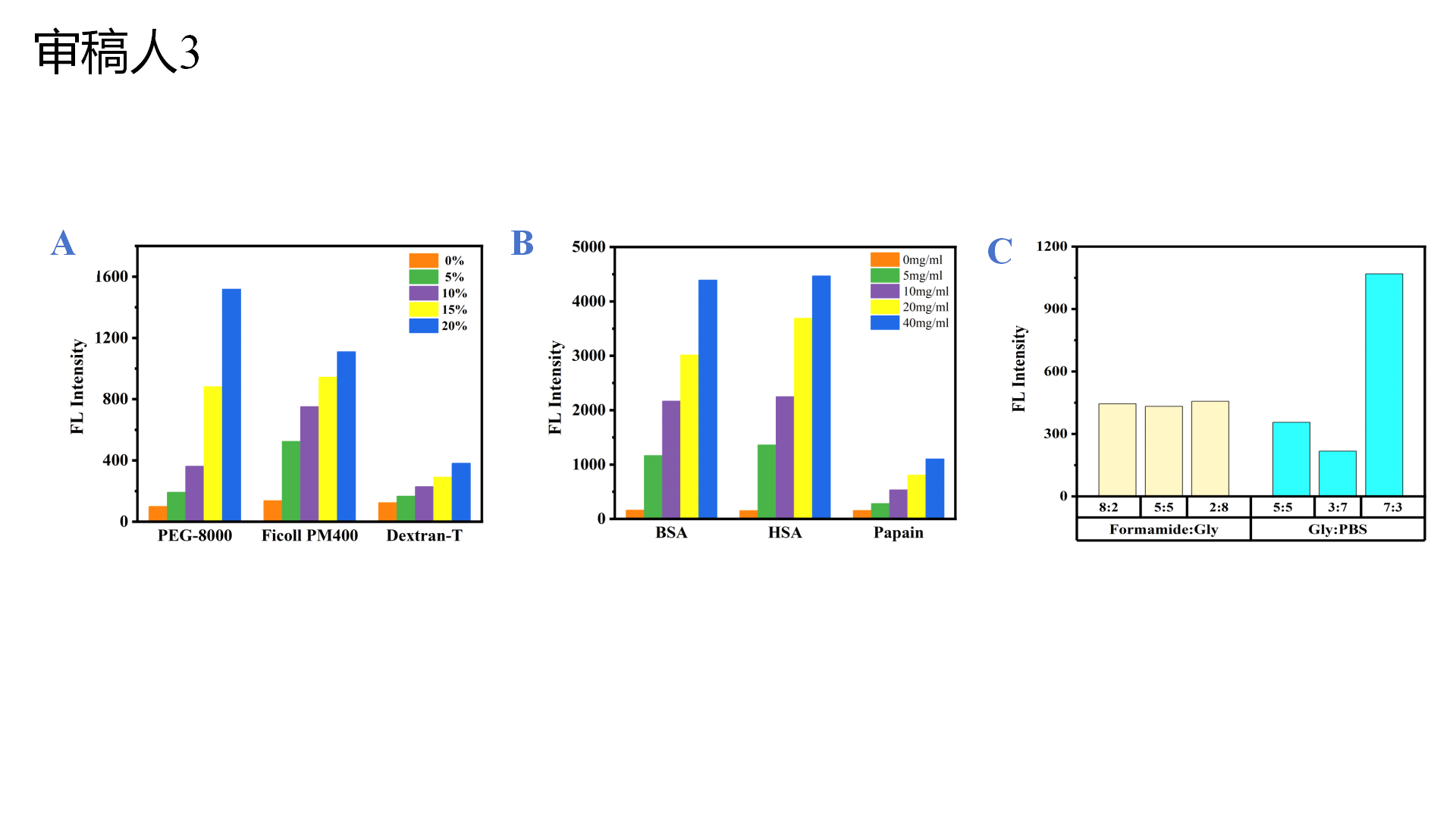


**Figure S16.** (A) Comparison of fluorescence intensity after mixing different concentrations of crowding/viscoelastic media (PEG/Ficoll/dextran-T) with 10μM probe **HJA-MQ-D**. (B) Comparison of fluorescence intensity after mixing three different protein concentrations with 10μM probe **HJA-MQ-D**. (C) Orthogonal experiment comparison. The excitation wavelength was 610 nm. Slits = 10/10 nm.

**Table S4.** Zeta Potential Analysis of Probe **HJA-MQ-D** Binding to BSA and HSA

|  | **BSA** | **HJA-MQ-D** | **BSA+HJA-MA-D** |
| --- | --- | --- | --- |
| **Zeta potential (mv)** | -15.36 | 5.049 | -14.05 |

|  | **HSA** | **HJA-MQ-D** | **HSA+HJA-MA-D** |
| --- | --- | --- | --- |
| **Zeta potential (mv)** | -7.664 | 5.509 | -5.354 |


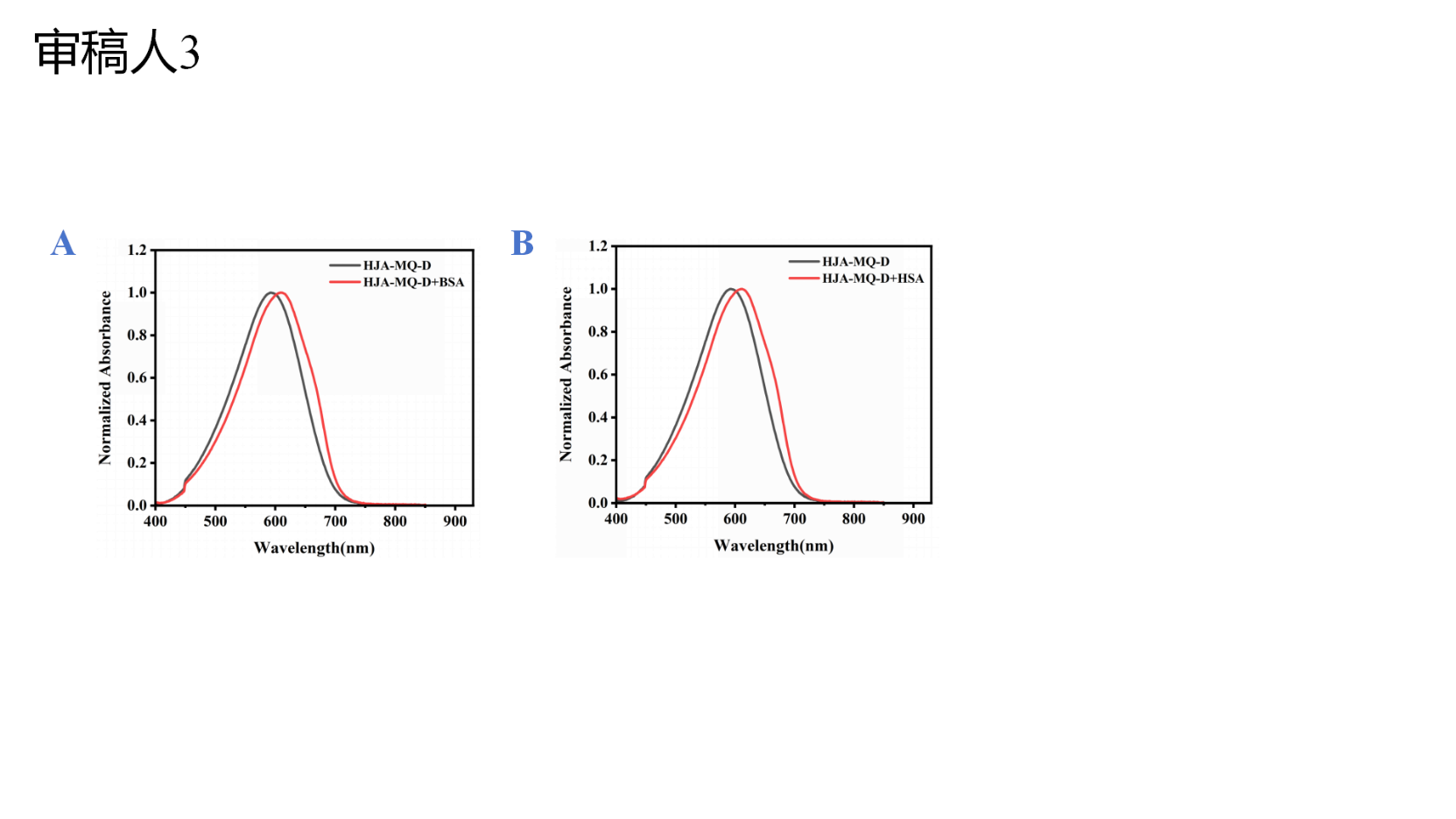


**Figure S17.** Ultraviolet Absorption Spectrum of Probe **HJA-MQ-D** after BSA/HSA Conjugation


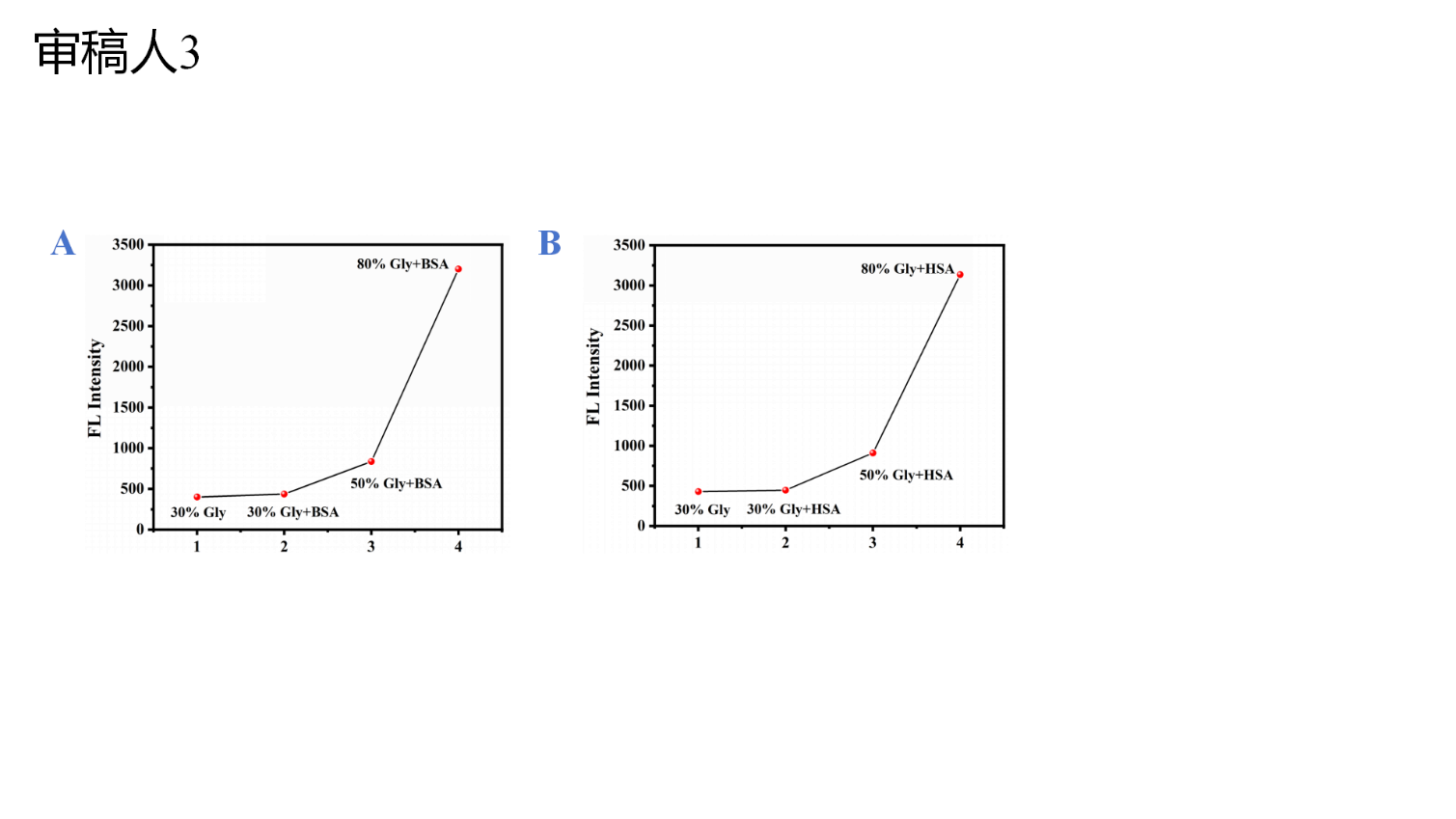


**Figure S18.** Fluorescence response of the probe **HJA MQ-D** in a viscosity-protein conjugate environment

1. **Analysis of Onion epidermal cells**

We first cut the inner epidermis of onions with a knife and tore off the epidermal tissue with forceps^29,30^, followed by staining with probe **HJA-MQ-D** (5 μM) for 5 min. In the first protocol, the stained epidermis was continuously immersed in 50 mM NaCl solution and sampled for imaging every 5 min (Figure S19A). The experimental results indicated that the fluorescence intensity of the onion inner epidermal cells increased with increasing NaCl concentration and treatment time, but the probe was gradually quenched when the immersion time exceeded 10 min, and the cells exhibited plasma wall separation. When the treatment was changed to sucrose solution, the fluorescence intensity was also enhanced with the increase of immersion time, but the quenching of the probe and plasmic wall separation were also observed after 10 min (Figure S19B). Additionally, we exposed the stained inner epidermal cells of onions to air to simulate drought stress (Figure S19C).


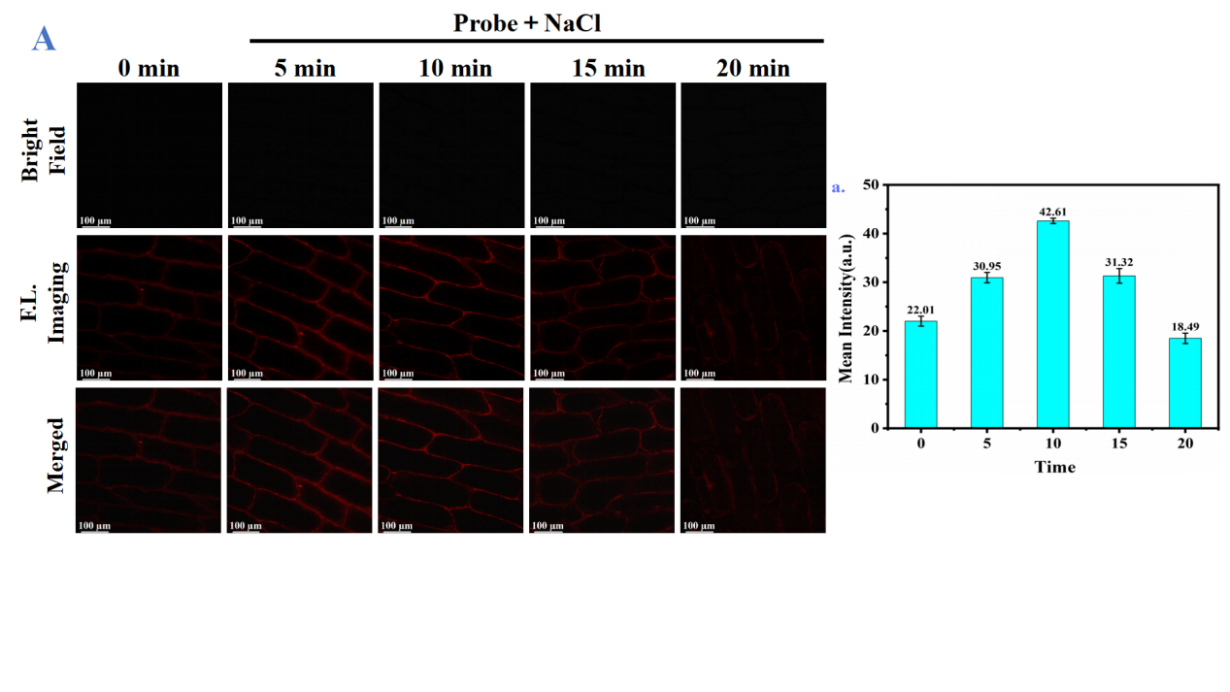


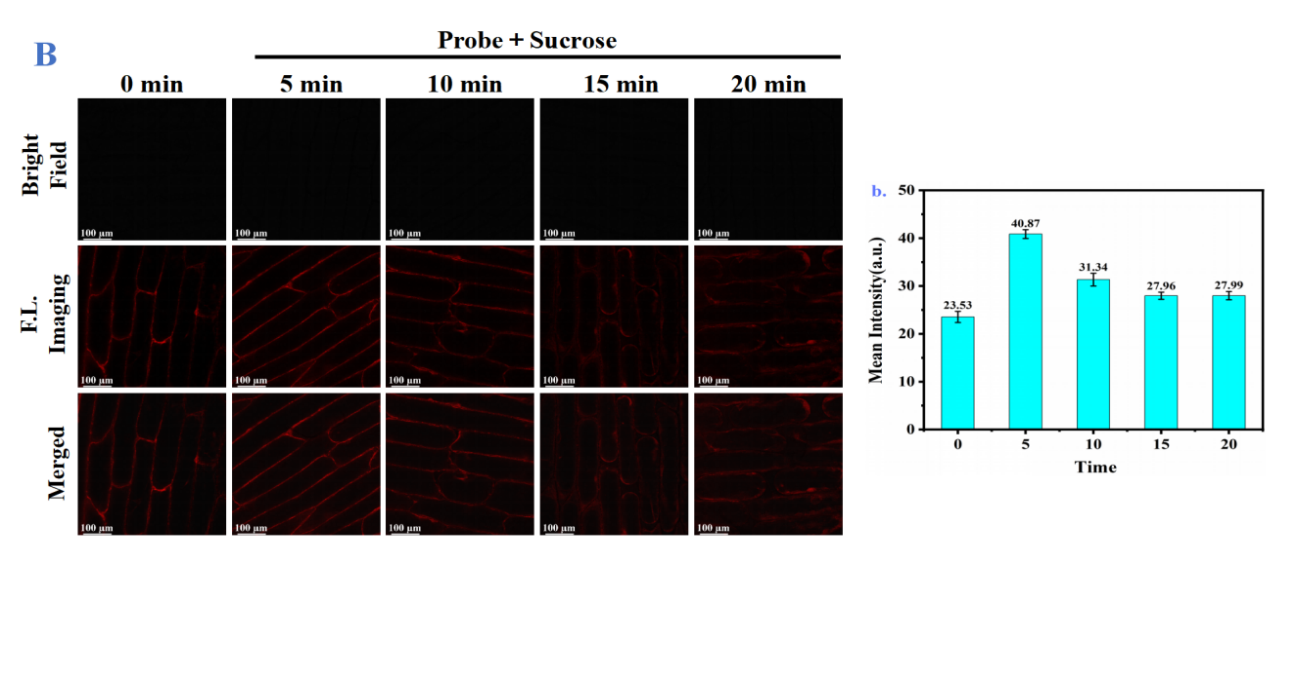


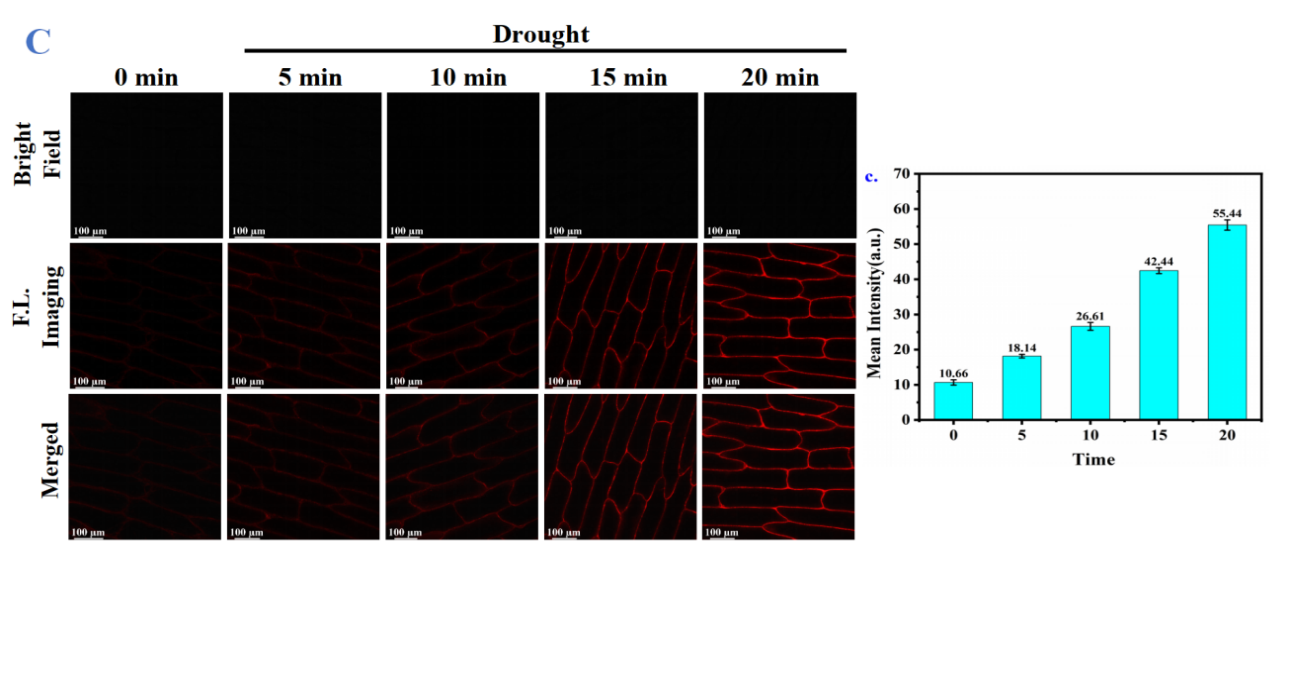


**Figure S19.** (A) Onion inner epidermis stained with 5 μM fluorescent probe for 5 min, then treated with 50 mM NaCl solution (imaging every 5 min). (a) Quantitative analysis of relative fluorescence intensity in red channel for (A). (B) Onion inner epidermis stained with 5 μM fluorescent probe for 5 min, then treated with 0.3 g/mL sucrose solution (imaging every 5 min). (b) Quantitative analysis of relative fluorescence intensity in red channel for (B). (C) Onion inner epidermis stained with 5 μM fluorescent probe for 5 min, then air-exposed (imaging every 5 min). (c) Quantitative analysis of relative fluorescence intensity in red channel for (C). Red channel: λ_ex_ = 638 nm, λ_em_ = 680-730 nm, Scale bar = 100 μm.

1. **Analysis of Mung bean slice cells**

An appropriate amount of mung beans was selected and sterilized with 75% ethanol for 30 seconds, then rinsed with sterile water for three times, followed by incubation in sterile petri dishes. Suitable humidity was maintained, and the water was changed daily during incubation until the mung beans germinated (Figure S20A). The germinated mung bean sprouts were taken and slices of uniform thickness were prepared using razor blades. The sections were first soaked in 5 μM probe solution for 5 min and imaged, then treated in 0.3 g/mL sucrose solution for 10 min and imaged again^31-33^. The experimental results indicated that the fluorescence intensity of the cells in the mung bean sprouts sections was significantly enhanced after treatment with sucrose solution due to the increase in cell viscosity (Figure S20B, b).


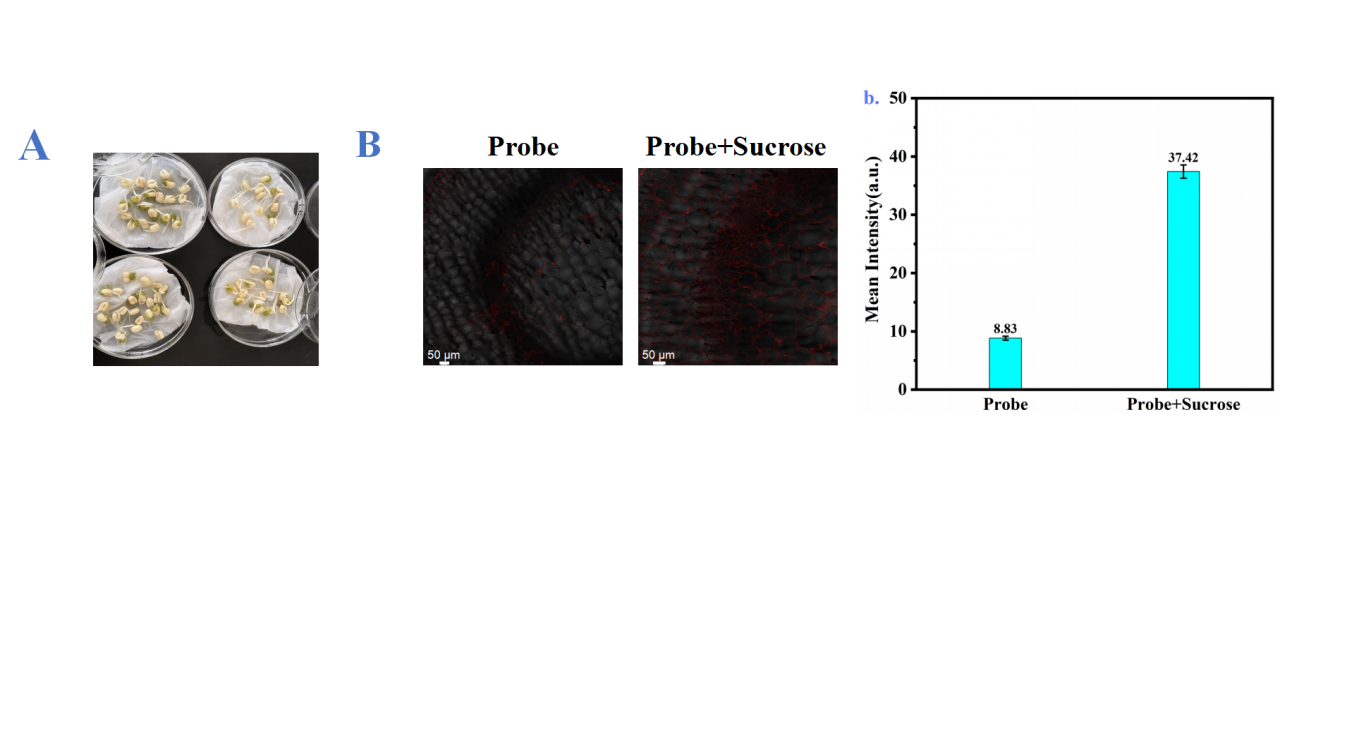


**Figure S20.** (A) A diagram of the mung bean germination culture process. (B) Microscopic images of mung bean seedling section cells from the control group (treated with 5 μM probe for 5 min) and experimental group (pretreated with 0.3 g/mL sucrose for 5 min followed by 5 μM probe for 5 min). (b) Quantitative analysis of relative fluorescence intensity in the red channel for (B). Red channel: λ_ex_ = 638 nm, λ_em_ = 680-730 nm, Scale bar = 50 μm.

1. **Mung Bean Roots**

Mung bean root cells labeled with the **HJA-MQ-D** probe were treated with salt concentration gradients (0, 30, 50, 100 mM NaCl). Confocal microscopy revealed that the fluorescence signal increased in a gradient manner with rising salt concentration, reaching its peak intensity in the 100 mM treatment group (Figure S21). The results confirm that salt stress rapidly induces an increase in intracellular viscosity in plant cells, and this probe can sensitively monitor this dynamic process.


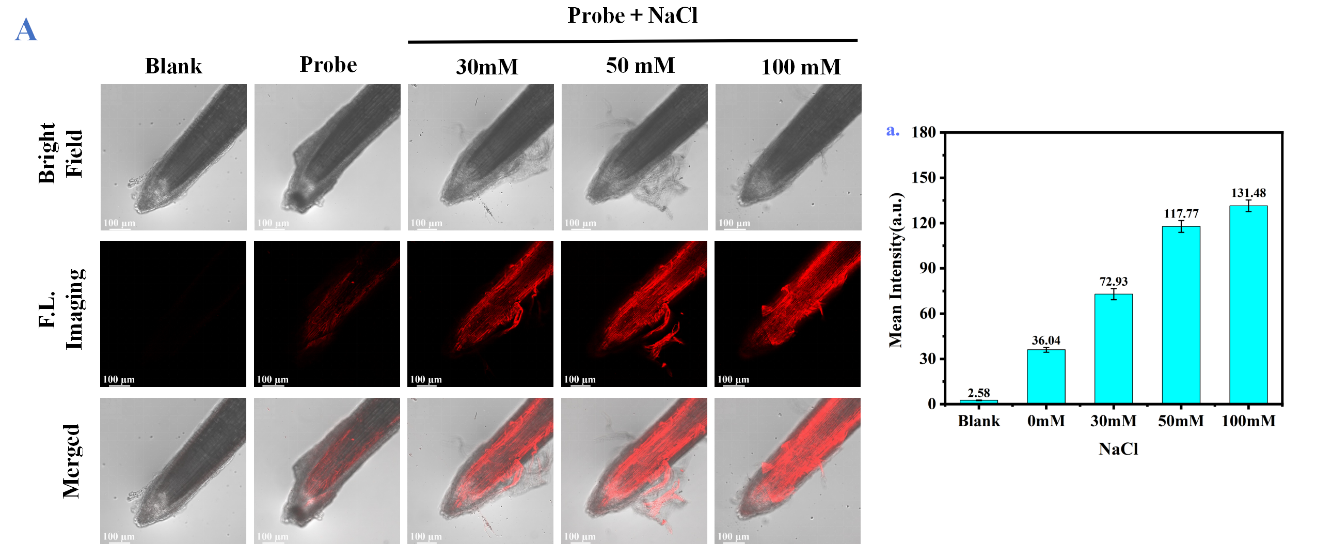


**Figure S21.** (A) Microscopic images of mung bean root cells from the control group (treated with 5 μM probe **HJA-MQ-D** for 5 min) and the experimental group (treated with 5 μM probe for 5 min after pretreatment with 0.3 g/mL sucrose for 5 min). (a) Quantitative analysis of relative fluorescence intensity in the red channel for (A). Red channel: λ_ex_ = 638 nm, λ_em_ = 680-730 nm, Scale bar = 100 μm.

1. **Analysis of probe HJA-MQ-D cytotoxicity in Hela cells**

HeLa cells were cultured in complete medium supplemented with 10% inactivated fetal bovine serum and incubated in a humidified incubator at 37 °C with 5 % CO₂ for 24 hours. The MTT assay was used to determine the cytotoxicity of probe **HJA-MQ-D** toward HeLa cells. HeLa cells were employed for confocal imaging (Figure S22).


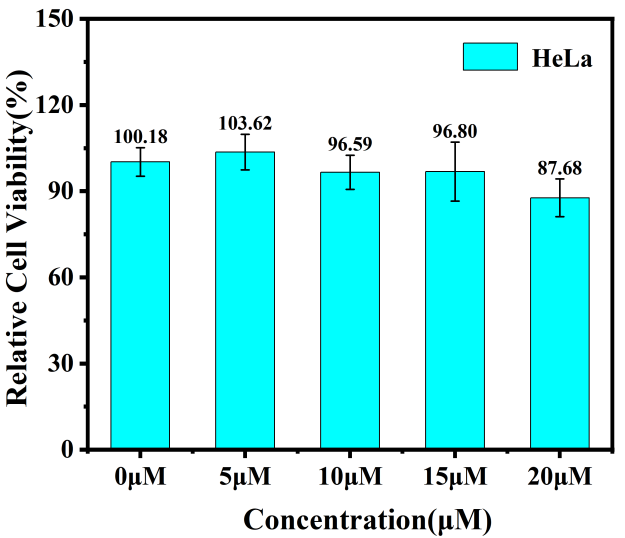


**Figure S22.** Cytotoxicity assays of probe **HJA-MQ-D** at different concentrations for HeLa cells.

1. **References**
2. D. Jia, Z. Li, H. Ma, H. Ji, H. Qi, C. Zhang, *Anal. Chem.* **2024**, *96* (15), 6030-6036.
3. H. Y. Zou, Z. C. Wang, X. H. Liu, Y. F. Du, X. Q. Cao, J. Shang, S. L. Shen, X. F. Zhang, *Spectrochim. Acta, A Mol. Biomol. Spectrosc.***2025**, *338*, 126175.
4. X. Wu, R. Zhang, Y. Li, Y. Gai, T. Feng, J. Kou, F. Kong, L. Li, B. Tang, *Anal. Chem.* **2023**, *95* (19), 7611-7619.
5. Y. M. Lin, Q. He, X. Y. Wang, F. F. Hua, X. Y. Liu, Y. L. Fu, *J. Agric. Food Chem.* **2023**, *71* (13), 5154-5161.
6. H. Wang, F. Cai, L. Zhou, J. He, D. Feng, Y. Wei, Z. Feng, X. Gu, U. Kajsa, Z. J. Hu, *New J. Chem.* **2019**, *43* (22), 8811-8815.
7. S. Cai, R. Guo, Q. Liu, X. Gong, X. Li, Y. Yang, W. J. Lin, *New J. Chem.* **2022**, *46* (17), 8171-8176.
8. X. Yin, Y. Cai, S. Cai, X. Jiao, C. Liu, S. He, X. Zeng, *RSC Adv.* **2020**, *10* (51), 30825-30831.
9. W. L. Cui, M. H. Wang, Y. H. Yang, X. Ji, J. Y. Wang, *Spectrochim. Acta, A Mol. Biomol. Spectrosc.* **2023**, *298*, 122775.
10. S. Erdemir, S. Malkondu, M. Oguz, O. Kocyigit, *Talanta* **2024**, *267*, 125143.
11. K. Zhou, M. Ren, B. Deng, W. J. Lin, *New J. Chem.* **2017**, *41* (20), 11507-11511.
12. S. Han, X. Jing, H. Peng, W. A. Lin, *Anal. Methods* **2025**, *17* (20), 4138-4149.
13. C. Peng, X. Ma, D. Lin, X. Feng, H. Yu, Y. Li, *Anal. Chim. Acta* **2021**, *1187*, 339146.
14. X. Wang, Z. Yin, H. Liu, Z. Wang, X. Zhu, Y. A. Ye, *J. Fluoresc.* **2025**, *35* (5), 2935-2942.
15. J. Liu, Y. Zhang, J. Hu, S. Chen, Y. Xue, W. Liu, L. Shen, A. Wang, Y. Zhu, L. Xu, *Spectrochim. Acta, A Mol. Biomol. Spectrosc.* **2026**, *345*, 126747.
16. H. Ren, X. Wang, Q. Pan, H. Wang, *Spectrochim. Acta, A Mol. Biomol. Spectrosc.* **2026**, *347*, 127046.
17. A. Maiti, S. Ahamed, M. Mahato, T. Ray, V. K. Dakua, K. Roy, M. N. Roy, *J. Fluoresc.* **2025**, *35* (2), 997-1010.
18. L. Zhao, H. Chu, S. Zhang, L. Xu, B. Yang, P. Ma, Q. Wu, D. Song, *Sens. Actuators B Chem.* **2023**, *375*, 132883.
19. Y. Zhao, L. Hu, T. Liang, M. Tian, C. Wu, L. Tang, X. Sun, Y. Li, X. Li, J. Li, K. Zhong, *Microchem. J.* **2025**, *212*, 113319.
20. J. Fu, S. He, J. Liu, J. Pang, K. N. Wang, Y. *J.* Chen, *Mater. Chem. B* **2024**, *12* (41), 10635-10643.
21. S. Taneja, K. Sharma, P. Selvam, S. K. A. Kumar, G. Thiruppathi, P. Sundararaj, S. K. Ramasamy, *J. Fluoresc.* **2025**, *35* (8), 6731-6745.
22. O. Batsaikhan, B. Choi, H. Nam, C. Kim, *Inorg. Chim. Acta* **2024**, *571*, 122234.
23. W. Luo, Q. Diao, L. Lv, T. Li, P. Ma, D. Song, *Sens. Actuators B Chem.* **2024**, *412*, 135805.
24. S. Zhang, Y. Zhang, L. Zhao, L. Xu, H. Han, Y. Huang, Q. Fei, Y. Sun, P. Ma, D. Song, *Talanta* **2021**, *233*, 122592.
25. M. Fu, W. Shen, Y. Chen, W. Yi, C. Cai, L. Zhu, Q. Zhu, *J. Mater. Chem. B* **2020**, *8* (6), 1310-1315.
26. R. Bhaskar, P. Somkuwar, M. Babu, M. Ann, V. Raju, S. A. Majeed, S. K. A. Kumar, *Synth. Met.* **2025**, *312*, 117870.
27. S. Sreejith, J. Joseph, M. Lin, N. V. Menon, P. Borah, H. J. Ng, Y. X. Loong, Y. Kang, S. W. Yu, Y. Zhao, *ACS Nano* **2015**, *9* (6), 5695-5704.
28. S. Metangle, N. Ranjan, *ChemBioChem* **2024**, *25* (3), e202300527.
29. A. J. Winstead, G. Nyambura, R. Matthews, D. Toney, S. Oyaghire, *Molecules* **2013**, *18* (11), 14306-14319.
30. W.-L. Cui, M.-H. Wang, Y.-H. Yang, X. Ji, J.-Y. Wang, *Spectrochim. Acta, A Mol. Biomol. Spectrosc.* **2023**, *298*, 122775.
31. X. Zhang, Z. Wang, H. Chu, Z. Li, Y. Xiong, Y. Chen, Q. Zhu, H. Feng, E. Zhu, J. Zhou, P. Huang, Z. Qian, *Anal. Chem.* **2022**, *94* (9), 4048-4058.
32. R. Chen, T. Hu, S. Xing, T. Wei, J. Chen, T. Li, Q. Niu, Z. Zhang, H. Ren, X. Qin, *Anal. Chim. Acta* **2023**, *1239*, 340735.
33. R. Chen, S. Xing, T. Hu, Y. Li, J. Chen, Q. Niu, T. Li, *Anal. Chim. Acta* **2023**, *1237*, 340557.
34. S. Erdemir, S. Malkondu, M. Oguz, O. Kocyigit, *Talanta* **2024**, *267*, 125143.
